# dsHMGB1, released from IL-17A-induced pyroptotic prostate epithelial cells, drives M1 polarization by promoting Pfkp-mediated glycolysis via Jak2/Stat1 transcription in experimental autoimmune prostatitis

**DOI:** 10.7150/ijbs.113908

**Published:** 2025-09-03

**Authors:** Wenming Ma, Yi Zhang, Wenlong Xu, Yongtao Hu, Weikang Wu, Lei Chen, Li Zhang, Hexi Du, Jialin Meng, Jing Chen, Chaozhao Liang

**Affiliations:** 1 Department of Urology, The First Affiliated Hospital of Anhui Medical University, Hefei, 230022, P.R. China.; 2 Institute of Urology, Anhui Medical University, Hefei, 230022, P.R. China.; 3 Anhui Province Key Laboratory of Urological and Andrological Diseases Research and Medical Transformation, Hefei, 230022, P.R. China.

**Keywords:** CP/CPPS, dsHMGB1, Jak2/Stat1, glycolysis, M1 polarization

## Abstract

**Background:** Chronic prostatitis/chronic pelvic pain syndrome (CP/CPPS) is a prevalent urological disorder in males, characterized by an unknown mechanism and limited therapeutic efficacy. The involvement of high mobility group box 1 (HMGB1)-mediated macrophage polarization has been extensively explored in various immune-inflammatory conditions; however, its potential role in CP/CPPS has not yet been examined.

**Method:** In experimental autoimmune prostatitis (EAP) mouse model, with various treatments including anti-IL-17A, Bz-ATP, or glycyrrhizic acid (GA, a HMGB1 inhibitor). In *vitro*, prostate epithelial cells (PECs) and immortalized bone marrow-derived macrophages (iBMDM) were treated with IL-17A, disulfide HMGB1 (dsHMGB1), or fludarabine (Flu, a Stat1 inhibitor). Histological analysis, immunofluorescence, TUNEL, ELISA, reactive oxygen species detection, glucose uptake, lactate assays, flow cytometry, western blot, proteome sequence, differential gene analysis, RT-qPCR, ChIP-qPCR, and dual-luciferase assay, etc. were used for the detection of phenotypes and exploration of mechanisms.

**Results:** We confirmed that IL-17A could induce pyroptosis in PECs and release dsHMGB1 in *vitro*, the similar function presented in *vivo* as well, and can be reversed by Bz-ATP. Additionally, dsHMGB1 enhances glycolytic metabolism via the Jak2/Stat1 pathway, thereby promoting polarization of M1 macrophage. Pfkp, a rate-limiting enzyme involved in glycolysis, plays a critical role in this metabolic shift. ChIP-qPCR and luciferase assays demonstrated that Stat1 can transcriptionally regulate Pfkp. In the rescue experiments, we also demonstrated that GA and Flu could potentially be the therapeutic options for CP/CPPS.

**Conclusions:** IL-17A-mediated pyroptosis in prostate epithelial cells triggers the release of dsHMGB1, which transcriptional regulates the key glycolytic enzyme Pfkp through the Jak2/Stat1 transcription to promote the M1 polarization of macrophages. Targeting dsHMGB1 or Stat1 could be potential therapeutic strategies for managing CP/CPPS by regulating M1 macrophage polarization and reducing inflammatory cytokines.

## 1. Introduction

Chronic prostatitis/chronic pelvic pain syndrome (CP/CPPS), categorized as type III prostatitis according to the National Institute of Health (NIH) classification system, represents about 90-95% of all prostatitis cases^1^. CP/CPPS classification is determined by the detection of white blood cells (WBCs) in post prostatic-massage urine or prostatic secretions, with type IIIa being inflammatory and type IIIb being noninflammatory^2^. Although CP/CPPS is not typically life-threatening, it is commonly associated with lower urinary tract symptoms (LUTS), erectile difficulties, and localized discomfort (such as perineal or testicular pain), all of which can seriously affect life^3^. The exact cause of CP/CPPS remains unclear, and as a result, no effective cure has been identified^4^. Current treatment approaches remain largely empirical, yielding suboptimal prognostic outcomes for patients^5^. Therefore, further investigation of the potential mechanism of CP/CPPS and the development of effective therapeutic strategies is crucial.

Previous studies have reported the presence of various immune cells in the prostate glands of individuals with CP/CPPS. Moreover, the immunopathogenic mechanisms related to immune cell infiltration (such as macrophage and T lymphocyte), have also been investigated for their potential role in the onset and progression of prostatitis^6^, ^7^. Immunization with prostate-associated antigens (PAgs) can elicit immune responses that drive the aberrant differentiation of T helper 17 (Th17) cells, thereby exacerbating prostate gland inflammation and chronic pelvic pain^8^, ^9^. Additionally, prophylactic treatment with antibodies targeting interleukin 17 (IL-17) has demonstrated efficacy in alleviating the typical symptoms of CP/CPPS^10^, ^11^. Despite these findings, the underlying mechanisms through which an elevated percentage of Th17 cells contributes to the development of CP/CPPS remain unclear, necessitating urgent and comprehensive investigation.

Pyroptosis is a type of regulated cell death characterized by inflammation, characterized by cell swelling and the discharge of intracellular components via membrane rupture^12^. Studies have shown that inflammasome activation facilitates the processing and release of the pro-inflammatory cytokines, along with the cleavage of Gasdermin D (GSDMD), thereby driving pyroptosis-mediated cell death^13^. Elevated IL-17A has been reported to induce pyroptosis in nasal epithelial cells (NECs) through the NLRP3/Caspase-1 pathway^14^. Moreover, Xuanbi yuyang decoction (XBD) has been shown to mitigate IL-17 induced pyroptosis in intestinal epithelial cells (IECs) and alleviate DSS-induced colitis^15^. Furthermore, IL-17A overexpression has been shown to promote pyroptosis in keratinocytes by antagonizing KRT6A^16^. Taken together, these results emphasize the essential involvement of IL-17A in inducing pyroptotic cell death within epithelial cells. However, whether IL-17A can induce pyroptosis in prostate epithelial cells (PECs) and subsequently influence the pathogenesis and progression of CP/CPPS needs further investigation.

High-mobility group box-1 (HMGB1) is a highly conserved chromosomal protein that exhibits a variety of functions based on its location within the cell^17^. HMGB1, a marker of pyroptosis, is upregulated through the NF-κB/NLRP3/GSDMD signaling pathway in IECs during pyroptosis^18^. In the context of infection, *S. pseudintermedius* has been shown to induce pyroptosis in canine corneal epithelial cells (CCECs), leading to the release of HMGB1^19^. Moreover, HMGB1 released by epithelial cells after pyroptosis has been implicated in promoting colorectal tumors^20^. Notably, the extracellular release of HMGB1 has the potential to activate macrophages, further intensifying local inflammation and exacerbating tissue damage^21^, ^22^. Given its pivotal role in aggravating inflammatory conditions, the extracellular functions of HMGB1 have attracted considerable attention, positioning it as a promising target for therapeutic intervention^23^. Typically, the redox status of three cysteine residues located at positions 23, 46 and 106 in HMGB1 plays a crucial role in modulating its extracellular inflammatory activity. Based on its redox state, HMGB1 exists in three distinct isoforms: disulfide HMGB1 (dsHMGB1), fully reduced HMGB1 (frHMGB1), and sulfonyl HMGB1 (oxHMGB1). Specifically, dsHMGB1 is the only subtype that has proinflammatory effects and plays an important role in inducing M1 macrophage polarization^24^, ^25^.

Macrophages significantly contribute to the onset and progression of chronic prostatitis. Our previous studies have demonstrated that CXCR4 enhances glycolysis, subsequently driving M1 macrophage polarization, which contributes to prostatic fibrosis and ultimately aggravate chronic prostatitis^26^. In addition, macrophage migration inhibitory factor (MIF) intensifies macrophage infiltration through the PI3K/Akt and NLRP3 pathways^27^. Moreover, CXCL10 facilitates macrophage migration and stimulates the release of inflammatory molecules by activating the ERK and p38 MAPK pathways via CXCR3, thus playing a pivotal role in promoting prostatic inflammation infiltration^6^. However, the precise contribution of M1 macrophage polarization to the pathogenesis and progression of CP/CPPS are not yet fully understood.

In this study, proteomic analysis of prostate tissues revealed the significant upregulation of HMGB1 in prostate tissue from experimental autoimmune prostatitis (EAP) model mice compared with control group. Further investigation suggested that IL-17A induces pyroptosis in PECs, leading to the release of dsHMGB1, which subsequently drives macrophage polarization toward the M1 phenotype, thus aggravating CP/CPPS. Mechanistic studies indicated that dsHMGB1 activates the Jak2/Stat1 pathway to regulate the transcription of Pfkp, thereby enhancing macrophage glycolysis and promoting M1 polarization.

## 2. Materials and methods

### 2.1 Mice preparation

The male NOD mice (5 weeks old) were bought from Biomedical Institute of Nanjing. The mice were maintained in a specific pathogen-free (SPF) laboratory animal facility at Anhui Medical University. All animal-related procedures were carried out following the guidelines outlined in the NIH guide for the care and use of laboratory animals, and approved by the Animal Welfare and Ethics Committee of Anhui Medical University (Approval No. LLSC20241770).

### 2.2 EAP mouse models establishment and treatment

The immunoreactive reagent, as previously described, was prepared by thoroughly emulsifying PAgs derived from round Sprague-Dawley (SD) rat prostate tissues with complete Freund's adjuvant (CFA, Sigma-Aldrich, USA) at a 1:1 ratio. Subsequently, male mice were subcutaneously injected with either 150 μl of the immunoreactive reagent or an equivalent volume of saline solution at multiple sites, including the shoulder (50 μl), the base of the tail (50 μl) and two foot pads (25 μl on each side), on days 0 and 14^28^. The pharmacological treatment of EAP was divided into three parts: (1) Control, EAP and EAP+anti-IL-17A groups: anti-mouse IL-17A (10 μg/mice, intraperitoneally (i.p.), Cat# MAB421, R&D System) was administered 1 day(d) before the first EAP model immunization and then once a week for four weeks in the EAP+anti-IL-17A groups^29^ ; (2) EAP+anti-IL-17A and EAP+anti-IL-17A+Bz-ATP groups: after i.p. of anti-IL-17A, Bz-ATP (5 mg/kg, Cat# GC15898, Glpbio) was administered for 14 consecutive days in the latter group^30^. (3) EAP and EAP+glycyrrhizicacid (GA) groups: after the second immunization, a 10-day consecutive treatment of GA (50mg/kg/d,i.p., Cat# HY-N0184, MCE) was administered to the EAP+GA group^31^, ^32^. Fourteen days after the second immunization, a von Frey test was performed to evaluate the mice's sensitivity to pelvic stimuli by applying a set of calibrated filaments. A positive pain reaction was defined as the occurrence of the following responses: (a) sudden contraction of the abdomen; (b) immediate licking or scratching of the stimulated area by filaments; (c) jumping^7^.

### 2.3 Cell culture and treatment

Mouse primary PECs were obtained from iCell Bioscience Inc (Shanghai) and cultured with iCell primary epithelial cell culture. Immortalized bone marrow derived macrophages (iBMDMs, presented by Academician Feng Shao) and HEK293T cells were cultured in high-glucose DMEM containing 10% FBS (Cat# BC-SE-FBS07, Biochannel) and 1% PS (Cat# C0222, Beyotime). IL-17A (Cat# HY-P73174) and fludarabine (Flu, Cat# HY-B0069) were purchased from MCE, and dsHMGB1 was purchased from HMGBiotech (HM-122, Milano). The cells were incubated with IL-17A (100 ng/ml) or dsHMGB1 (5 μg/ml), or Flu (100 μM, added 1 h before dsHMGB1 stimulation) in serum-free medium for 24 h. Gene knockdown was achieved via small interfering RNA (siRNA) transfection. Before beginning the procedure, the cells were cultured in 6-well plates and permitted to attach, reaching about 50-70% at the time of transfection. The serum-free culture medium was replaced 30 minutes prior to transfection. A mixture comprising 100 nM siRNA and 2.4 µM transfection reagent was formulated and left to stand at normal temperature for 15 min, and then add it to the culture medium. Following an incubation period of 24 h, the serum-free medium was replaced and further processing was carried out. The sequences of primers used to prepare these siRNAs are provided in **Table S1**.

### 2.4 Proteome sequencing and analysis

The proteomic sequencing process involves several key steps, including extraction of proteins, denaturation, restoring alkylation, enzyme hydrolysis, and peptide desalination. These procedures were performed on mouse prostate tissues obtained from both the control and EAP groups. Additionally, a database was established for data collection, along with subsequent qualitative and quantitative analyses of proteins. To determine proteins that showed notable variations in abundance across the control and EAP groups, a differential protein analysis was conducted using the quantitative data obtained. Proteins were considered to exhibit significant differences in expression if they met the criteria of *P* < 0.05 and fold change > 1.5 or < 1/1.5. The proteomics data generated in our study are novel and have been fully uploaded to a publicly accessible repository. The raw and processed proteomics data can be accessed at iProx with the accession code [IPX0012667000, PXD066293]^33^, ^34^.

### 2.5 Hematoxylin-Eosin (H&E) staining

Mouse prostate tissues were collected and preserved in 4% paraformaldehyde. Subsequently, histopathology was conducted using H&E staining. The staining process involved several key steps, including deparaffinization, staining, dehydration, tissue clearing, and finally sealing. To evaluate the extent of inflammation, a previously established grading system with scores ranging from 0-3 was employed: 0, no inflammatory response observed; 1, mild inflammation with clear perivascular cuffing containing mononuclear cells; 2, moderate perivascular cuffing with infiltration of mononuclear cells; and 3, severe perivascular cuffing accompanied by hemorrhage and extensive mononuclear cell infiltration in the parenchymal tissue^35^, ^36^.

### 2.6 Immunofluorescence (IF) staining

After deparaffinization of the paraffin sections, antigen retrieval was carried out by EDTA, after which the sections were rinsed to eliminate residual liquid and prepared for antigen blocking. The sections were left to incubate with the corresponding primary antibody at a temperature of 4°C overnight. The sections were then treated with the secondary antibody and incubated for 2 h in the dark at room temperature. Following this, the tissue sections were labeled with DAPI stain and kept in a dark environment at room temperature for 10 minutes. Finally, after the sections were sealed, they were observed under a fluorescence microscope. The antibodies utilized are detailed in **Table S2**.

### 2.7 Immunohistochemical (IHC) staining

Prostate tissue sections were prepared according to the H&E and IF staining methods described above. After fixation, permeabilization and incubation with primary antibodies, on the second day, incubation was continued for another 2 hours with secondary antibodies. Subsequently, the observation was conducted under a fluorescence microscope. The antibodies are listed in **Table S2**.

### 2.8 TUNEL assay

TUNEL staining was conducted using a TUNEL assay kit following the manufacturer's protocol (Cat# RCT-50G, RecordBio). In brief, section was incubated with a preprepared detection reagent. The fluorescence signal was detected using a fluorescence microscope as previously described.

### 2.9 ELISA assay

The IL-17A (Cat# MK5818A, Meike) and dsHMGB1 (Cat# MK0058MA, Meike) were measured in the serum and prostate tissues of the mouse. Additionally, the concentrations of dsHMGB1 were determined in the supernatant of PECs subjected to various treatments.

### 2.10 Determination of reactive oxygen species (ROS)

iBMDM cells were cultured with plate-bound dsHMGB1 (5 μg/mL) or Flu (100 μM, added 1 h before dsHMGB1 stimulation) for 24 h. The cells were incubated with DCFH-DA (Cat# S0033S, Beyotime, 10 μM) at 37°C for 20 min. Intracellular ROS levels were then assessed by detecting DCF fluorescence using fluorescence microscope.

### 2.11 Glucose uptake assay

The macrophages were treated according to the above experimental plan for 24 h, harvested into flow tubes and incubated at 37°C (dark condition) with 100 μM of 2-NBDG (Cat #GC10289, Glpbio) for 30 minutes. After two washes, the mean fluorescence intensity was detected by flow cytometer (Beckman, USA).

### 2.12 Lactate assay

Lactate levels in cells were quantified employing a lactate detection kit (Cat# KTB1100, Abbkine) according to the manufacturer's guidelines.

### 2.13 Flow cytometry

The polarization ratio of macrophages was determined by flow cytometry. Mouse spleen macrophages and iBMDM were incubated with labeled antibody that specifically targets CD11b (Cat# 101212, Biolegend; Cat# 557396, BD Bioscience), F4/80 (Cat# 746070, BD Bioscience), MHC II (Cat#107605,107607, Biolegend) or CD206 (Cat# 565250, BD Bioscience) for 1 h at room temperature. For flow cytometry analysis of the macrophage in spleens of EAP mouse, we first delineated live cells with FSC-A/SSC-A, then removed the adhesion (P1-2), and finally selected F4/80 and CD11b positive cells (P2-4) as macrophages. On this basis, we specifically identified the MHC II positive cells (P4-5) as M1 polarized cells (**Figure S1A**). In the flow cytometry analysis of iBMDMs, the initial step involves identifying live cells on the basis of their FSC-A and SSC-A characteristics. Following this, cell adhesion is excluded through gating (P1-2). Subsequently, macrophages are defined by selecting the CD11b-positive population (P2-3). Within this macrophage subset, the M1 and M2 polarization states are determined by the expression of specific markers: MHC II positivity (P3-4) indicates M1 polarization, whereas CD206 positivity (P3-4) identifies M2-polarized macrophages (**Figure S1B-C**). The stained cells were examined employing a flow cytometer with CytExpert software.

### 2.14 Western blotting

The protein was isolated through RIPA, PMSF, phosphatase and protease inhibitors. The proteins were then separated and transferred to a PVDF membrane (Millipore, Ireland). The membrane was blocked for 1h, incubated with the primary antibody at 4°C overnight and secondary antibody for 1h. The antibodies utilized are listed in **Table S2**.

### 2.15 RT-qPCR

The RNA was isolated utilizing an RNA extraction kit (Cat# 220011, Fastagen), cDNA was synthesized using an All-in-one RT EasyMix for qPCR Kit (Cat# 22107, Tolobio). RT-qPCR was conducted using SYBR master mix (Cat# 22204, Tolobio) and ddH_2_O. The primers are listed in **Table S3**.

### 2.16 Differentially expressed gene (DEG) analysis

To identify DEGs in the analysis of gene expression, we utilized the DESeq2 package and considered genes with *P* < 0.05 and |log_2_Fc| > 2 as significant. The transcriptomic data can be found in the GEO database under the assigned GEO accession number GSE200210^24^.

### 2.17 Chromatin immunoprecipitation qPCR (ChIP-qPCR)

ChIP-qPCR was performed following the instructions provided with the kit. Immunoprecipitation was conducted with an anti-Stat1 antibody (1:50, Cat# 9172T, CST). Furthermore, the purified DNA was subjected to qPCR analysis. The primers are listed in **Table S4**.

### 2.18 Dual-luciferase reporter assay

To confirm whether Pfkp is transcriptionally regulated by Stat1, we inserted the Pfkp promotor into the pGL3 vector and constructed a reporter gene vector. Additionally, for the expression of the target gene, we cloned the TF into the pcDNA3.1 expression vector. HEK293T cells were transfected with the reporter gene, target gene, and PRL-TK vectors in the experimental group, while the empty control, reporter gene, and PRL-TK vectors were transfected in the control group. Cell lysis was performed for detection using a luminometer to measure the luciferase and Renilla signals after incubation.

### 2.19 Seahorse XF assays

A total of 1.2 × 10^4 iBMDMs were seeded into each well of an Agilent seahorse XFe96/XF Pro cell culture microplate. Following a 1h incubation at room temperature on a sterile bench to allow cell attachment, the cells were treated overnight with dsHMGB1 or Flu mentioned above. Probe plates containing sterile water and calibration solution were simultaneously incubated overnight at 37 °C in a non-CO_2_ incubator. After overnight incubation, probe plates underwent calibration preparation: sterile water was discarded, 200 μl calibration solution was added, and plates were incubated (37 °C, non-CO₂) for 1h. Glycolysis stress test medium (supplemented with 2 mM glutamine) was subsequently mixed as an independent preparation. Following washing with designated test medium, plates were transferred to a cell culture incubator (37 °C, non-CO₂) for 1h. Glycolysis stress testing utilized 10 mM glucose, 1.0 μM oligomycin, and 50 mM 2-deoxyglucose (2-DG). Initiate by calibrating the probe plate using the Seahorse XF Pro Analyzer (Agilent). Upon completion, switch the hydration plate for the cell culture plate and conduct real-time metabolic analysis on the XFe96 analyzer, with data processed via Wave software.

### 2.20 Statistical analysis

Data analysis was conducted employing GraphPad Prism (v10.1), SPSS (v26.0), and R software (v4.2.3). Group differences were evaluated employing two-tailed Student's t-tests, ANOVA, or the Wilcoxon rank-sum test. Statistical significance was defined as a *P*-value less than 0.05.

## 3. Results

### 3.1 HMGB1 is upregulated in EAP mouse

**Figure 1:**
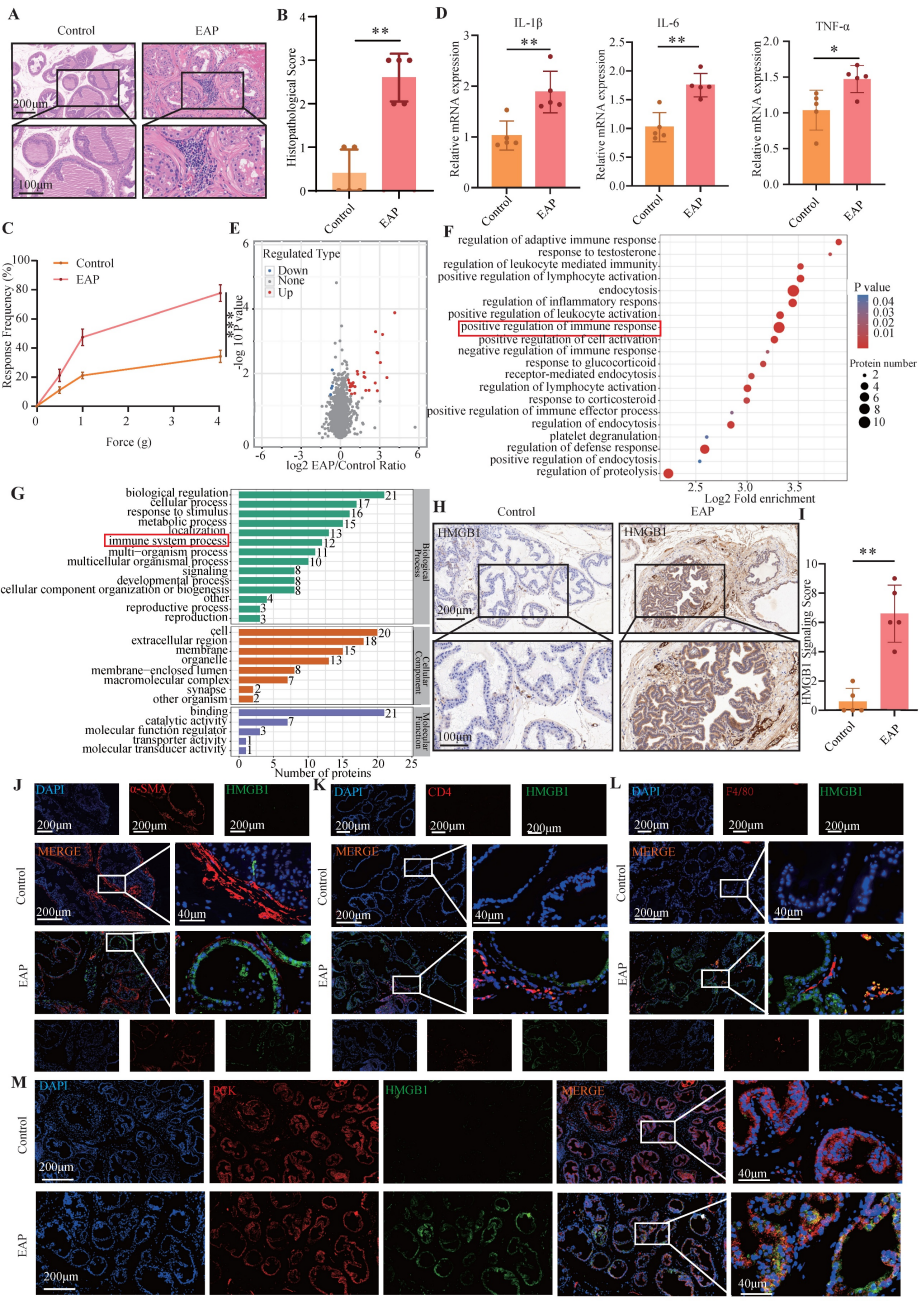
**HMGB1 is up-regulated in EAP mice.** (A-C) HE staining, inflammation score and pain response frequency in control and EAP groups of mice; (D) The RNA levels of inflammatory factors IL-1β, IL-6 and TNF-α between control and EAP groups were detected by RT-qPCR; (E-G) Proteomics sequencing analysis; (H-I) The expression levels of HMGB1 among the control and EAP groups were detected by immunohistochemical staining; (J-M) The co-immunofluorescence staining of HMGB1 with α-SMA, CD4, F4/80 and PCK. *P < 0.05; **P < 0.01; ***P < 0.001.

We successfully established an EAP model, as demonstrated by the significant accumulation of immune cells in the EAP group, but not in the control group (**Figure 1A-B**), as well as the increased response frequency of pain behavior test (**Figure 1C**). RT-qPCR revealed that the expression of IL-1β, IL-6, and TNF-α were elevated in the EAP group (all *P* < 0.05, **Figure 1D)**. We further performed proteomics sequencing and revealed that several proteins, especially HMGB1, were dramatically upregulated in EAP mouse, while immune-related response and process pathways also emerged from the enrichment analysis (**Figure 1E-G**). Further immunohistochemical staining confirmed that HMGB1 was significantly upregulated in the EAP group (**Figure 1H-I**). To investigate the localization of HMGB1 in prostate tissue, co-immunofluorescence staining of HMGB1 with α-SMA, CD4, F4/80 and PCK was performed. The findings demonstrated that HMGB1 was predominantly localized in the prostatic epithelium (**Figure 1J-M**).

### 3.2 dsHMGB1 released from IL-17A-induced pyroptotic prostate epithelial cells

**Figure 2:**
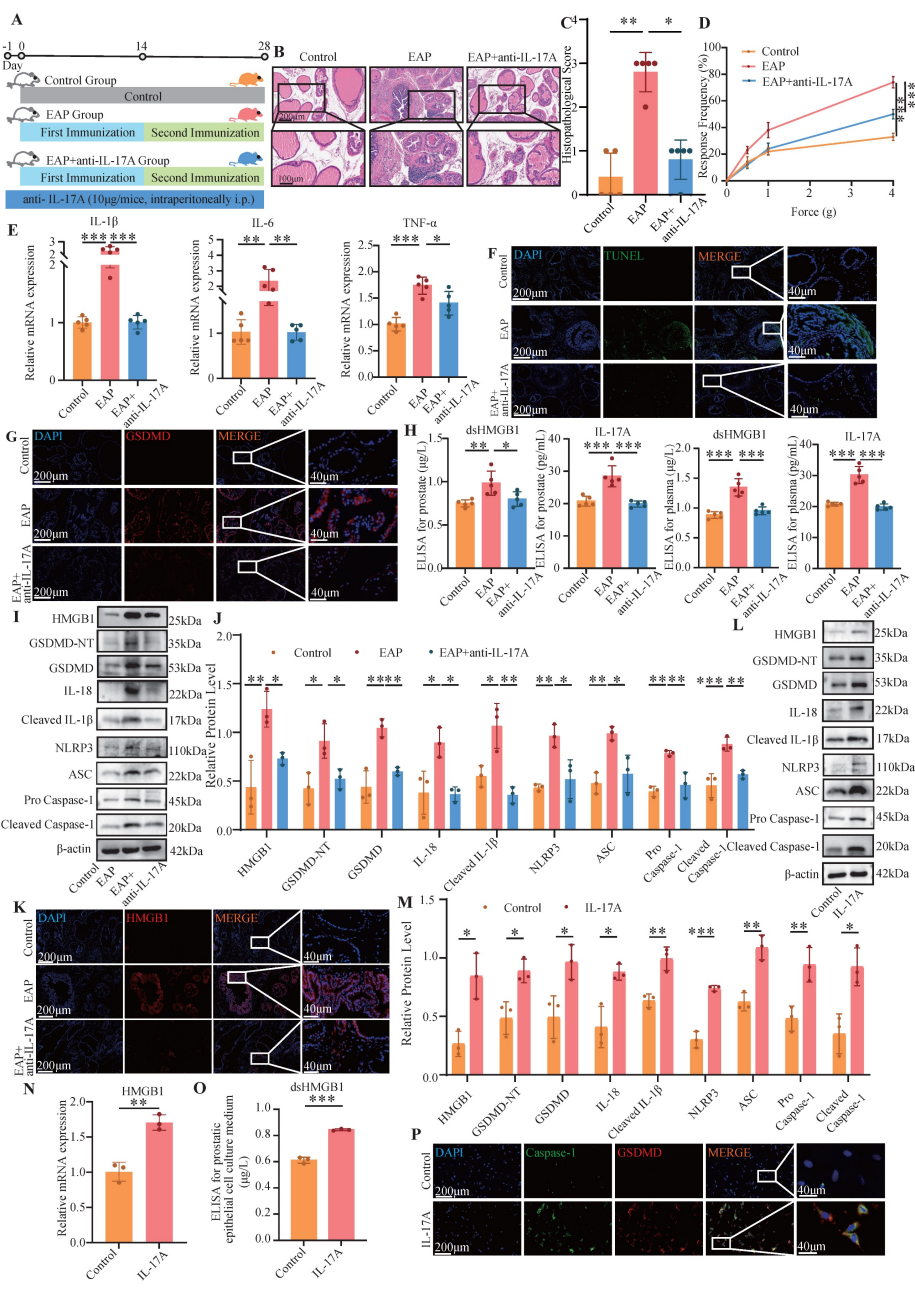
** dsHMGB1 released from IL-17A-induced pyroptotic prostate epithelial cells.** (A-D) The flow chart, HE staining, inflammation score and pain response frequency in control, EAP and EAP+anti-IL-17A groups of mice; (E) The RNA levels of inflammatory factors IL-1β, IL-6 and TNF-α between control, EAP and EAP+ anti-IL-17A groups were detected by RT-qPCR; (F-G) The TUNEL and GSDMD staining between control, EAP and EAP+anti-IL-17A group; (H) The levels of IL-17A and dsHMGB1 among the control, EAP and EAP+anti-IL-17A groups was detected by ELISA; (I-J) The expression levels of HMGB1, GSDMD-NT, GSDMD, IL-18, Cleaved IL-1β, NLRP3, ASC, Pro Caspase-1 and Cleaved Caspase-1 among the control, EAP and EAP+anti-IL-17A groups was detected by western blot; (K) The immunofluorescence staining of HMGB1 among the control, EAP and EAP+anti-IL-17A groups; (L-M) The expression levels of HMGB1, GSDMD-NT, GSDMD, IL-18, Cleaved IL-1β, NLRP3, ASC, Pro Caspase-1 and Cleaved Caspase-1 among the control and IL-17A groups was detected by western blot; (N) The RNA levels of HMGB1 between control and IL-17A groups were detected by RT-qPCR; (O) The levels of dsHMGB1 between control and IL-17A groups were detected by ELISA; (P) The co-immunofluorescence staining of Caspase-1 and GSDMD between control and IL-17A groups. *P < 0.05; **P < 0.01; ***P < 0.001.

In the next step, we applied an anti-IL-17A antibody to the EAP model and observed that it alleviated prostate inflammation and the response frequency of the pain behavior test (**Figure 2A-D**). The RT-qPCR revealed that the expression of IL-1β, IL-6 and TNF-α in the EAP group was significantly greater than that in both the control group and the EAP+anti-IL-17A group (all *P* < 0.05, **Figure 2E**). TUNEL and IF staining revealed that, compared with the other two groups, the EAP group exhibited severe pyroptosis (**Figure 2F-G**). ELISA further confirmed the significant increase of dsHMGB1 and IL-17A expression in the EAP group compared with the control group, which was decreased with anti-IL-17A treatment (all *P* < 0.05, **Figure 2H**). Furthermore, Western blot and IF analyses were conducted to assess the expression of pyroptosis-related proteins and HMGB1 in prostate tissues from the three groups. The results revealed significant upregulation of both HMGB1 and pyroptosis-related proteins in the EAP group compared with the control group, while it was decreased in the EAP+anti-IL-17A group (all *P* < 0.05, **Figure 2I-K**). Given that IL-17A is known to induce pyroptosis in PECs and that HMGB1 serves as an important marker for pyroptosis, we hypothesize that IL-17A might facilitate the release of HMGB1 by inducing pyroptosis in PECs.

In further experiments, we stimulated PECs with IL-17A and performed a series of assays, including RT-qPCR, Western blotting, ELISA and IF. The results consistently demonstrated an elevated HMGB1 expression alongside pyroptosis-related proteins, providing strong evidence that IL-17A triggers pyroptosis, leading to the release of dsHMGB1 in PECs (**Figure 2L-P**).

### 3.3 Bz-ATP reversed pyroptosis which was alleviated by anti-IL-17A

**Figure 3:**
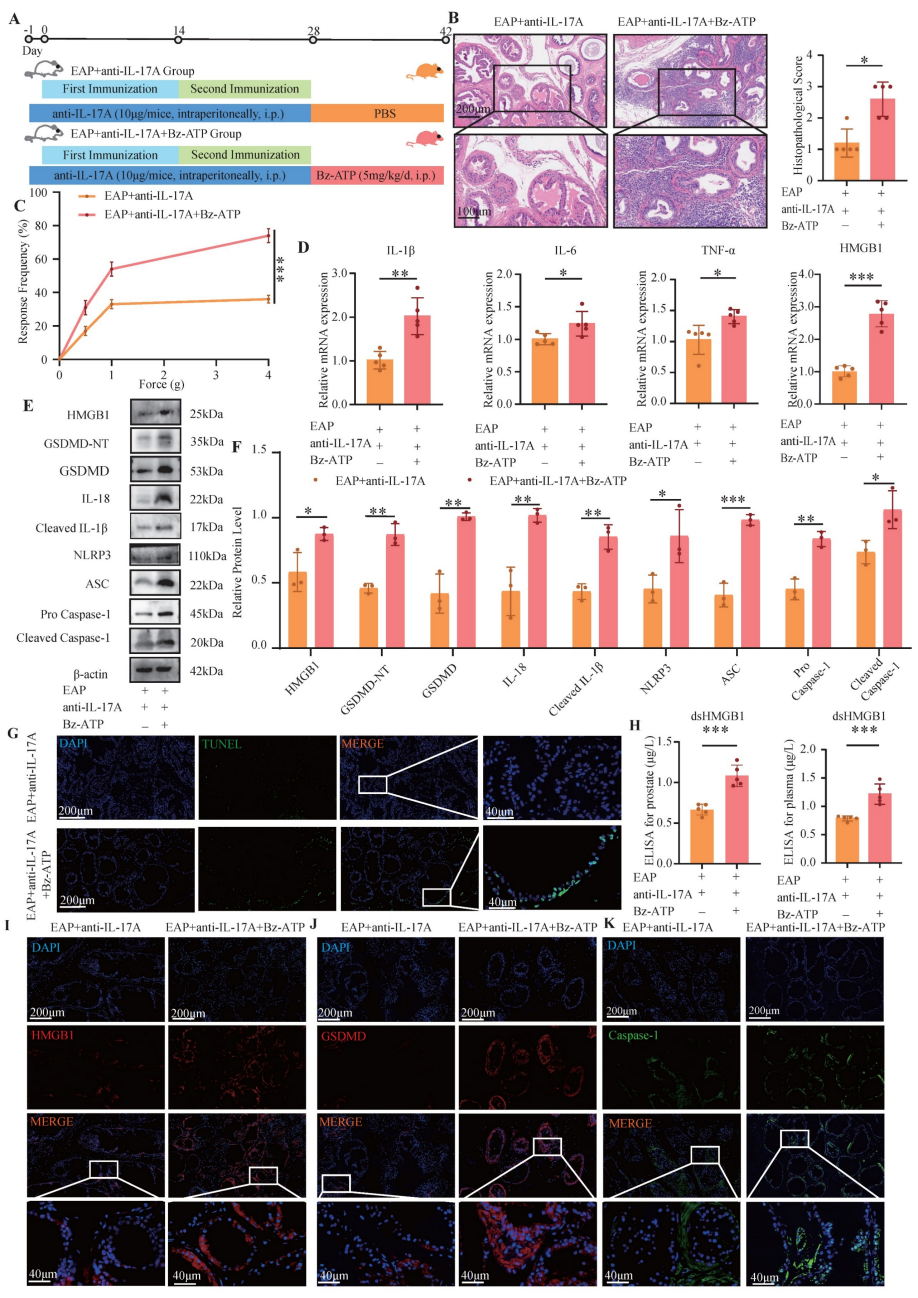
** Bz-ATP reversed pyroptosis which was alleviated by anti-IL-17A.** (A-C) Flow chart, HE staining, inflammation score and pain response frequency of prostate tissue in EAP+anti-IL-17A and EAP+anti-IL-17A+Bz-ATP groups of mice. (D) The RNA levels of inflammatory factors IL-1β, IL-6, TNF-α and HMGB1 in EAP+anti-IL-17A and EAP+ anti-IL-17A+Bz-ATP groups by RT-qPCR; (E-F) The protein levels of HMGB1, GSDMD-NT, GSDMD, IL-18, Cleaved IL-1β, NLRP3, ASC, Pro Caspase-1, and Cleaved Caspase-1 between EAP + anti-IL-17A and EAP+ anti-IL-17A+Bz-ATP groups were detected by western blot; (G) The TUNEL staining between EAP+anti-IL-17A and EAP+anti-IL-17A+Bz-ATP group; (H) The levels of dsHMGB1 among the EAP+anti-IL-17A and EAP+anti-IL-17A+Bz-ATP groups was detected by ELISA; (I-K) The expression levels of HMGB1, GSDMD and Caspase-1 among the EAP+anti-IL-17A and EAP+anti-IL-17A+Bz-ATP groups were detected by immunofluorescence staining. *P < 0.05; **P < 0.01; ***P < 0.001.

The response frequency in the pain behavior test and prostate tissue inflammation score of EAP mice were significantly increased after injection of anti-IL-17A followed by Bz-ATP treatment (*P* < 0.05, **Figure 3A-C**). The RT-qPCR results revealed increased expression of HMGB1, IL-1β, IL-6, and TNF-α in the EAP+anti-IL17A+Bz-ATP group (all *P* < 0.05, **Figure 3D**). The pyroptosis-related proteins and HMGB1 in the EAP+anti-IL-17A+Bz-ATP group was significantly greater than that in the EAP+anti-IL-17A group (all *P* < 0.05, **Figure 3E-F**). TUNEL staining revealed more severe apoptosis in the EAP+anti-IL-17A+Bz-ATP group than in the EAP+anti-IL-17A group (**Figure 3G**). ELISAs revealed that the expression of dsHMGB1 in the EAP+anti-IL-17A+Bz-ATP group was greater than that in the EAP+anti-IL-17A group (all *P* < 0.05, **Figure 3H**). IF also revealed that HMGB1 and pyroptosis-related proteins in the EAP+anti-IL-17A+Bz-ATP group was greater than that in the EAP+anti-IL-17A group (all *P* < 0.05, **Figure 3I-K**).

### 3.4 GA and anti-IL-17A inhibited M1 polarization, whereas Bz-ATP promoted M1 polarization

**Figure 4:**
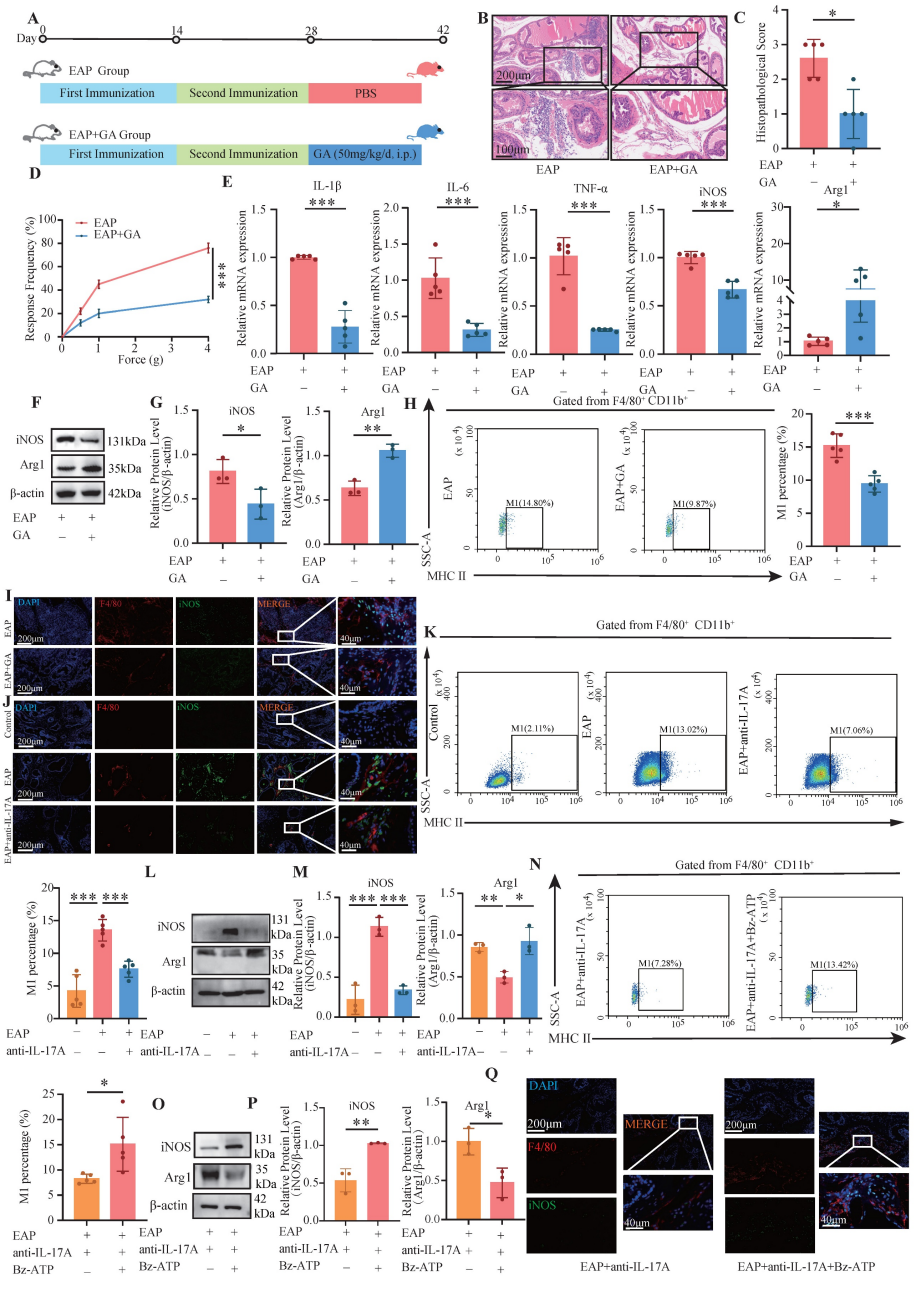
** GA and anti-IL-17A inhibited M1 polarization, while Bz-ATP promoted M1 polarization.** (A-D) Flow chart, HE staining, inflammation score and pain response frequency of prostate tissue in EAP and EAP+GA groups of mice; (E) The RNA levels of inflammatory factors IL-1β, IL-6, TNF-α, iNOS and Arg1 in EAP and EAP+GA groups by RT-qPCR; (F-G) The protein levels of iNOS and Arg1 between EAP and EAP+GA groups were detected by western blot; (H) Flow cytometry was used to detect the M1 polarization of macrophages between EAP and EAP+GA groups; (I) The IF staining of F4/80 and iNOS between EAP and EAP+GA groups; (J) The IF staining of F4/80 and iNOS between control, EAP and EAP+anti-IL-17A group; (K) Flow cytometry of the M1 polarization of macrophages between control, EAP and EAP+anti-IL-17A groups; (L-M) The protein levels of iNOS and Arg1 between control, EAP and EAP +anti-IL-17A groups were detected by western blot; (N) Flow cytometry of the M1 polarization of macrophages between EAP+anti-IL-17A and EAP+anti-IL-17A+Bz-ATP groups; (O-P) The protein levels of iNOS and Arg1 between EAP+anti-IL-17A and EAP+anti-IL-17A+Bz-ATP groups were detected by western blot; (Q) The IF staining of F4/80 and iNOS between EAP+anti-IL-17A and EAP+anti-IL-17A+Bz-ATP groups. *P < 0.05; **P < 0.01; ***P < 0.001.

GA, the primary bioactive component of licorice, is widely recognized for its pharmacological properties, including anti-inflammatory and antioxidant effects. Studies have demonstrated that GA can suppress the release of HMGB1 from damaged cells^37^, ^38^. The administration of GA led to a notable decrease in the pain response frequency and a reduction in prostate tissue inflammation (all *P* < 0.05, **Figure 4A-D**). The RT-qPCR results revealed that, compared with those in the EAP+GA group, the expression levels of IL-1β, IL-6, TNF-α, and iNOS in the EAP group were significantly increased. Conversely, Arg1 expression was significantly downregulated in the EAP group (all *P* < 0.05, **Figure 4E**). Western blot analysis also revealed that iNOS was significantly down-regulated in the EAP+GA group, whereas Arg1 was significantly up-regulated (all *P* < 0.05, **Figure 4F-G**). These findings suggest that GA can alleviate prostate tissue inflammation and inhibit M1 macrophage polarization in EAP mice. In addition, flow cytometry analysis also revealed that GA inhibited the M1 polarization of macrophages in EAP mice (*P* < 0.05, **Figure 4H**). The IF results further confirmed that GA alleviated the M1 polarization of EAP mouse macrophages (**Figure 4I**). Moreover, anti-IL-17A inhibited the M1 polarization of EAP mouse macrophages, whereas Bz-ATP reversed the suppressed M1 polarization (**Figure 4J-Q**).

### 3.5 dsHMGB1 promotes glycolysis-dependent M1 macrophage polarization

Previous studies have identified three redox isoforms of HMGB1: dsHMGB1, frHMGB1, and oxHMGB1. Among these, only dsHMGB1 has been shown to induce M1 macrophage polarization. Following the experimental approach outlined in prior research, dsHMGB1 was utilized to stimulate macrophages and a series of validations confirmed that dsHMGB1 can induce M1 macrophage polarization^24^.

**Figure 5:**
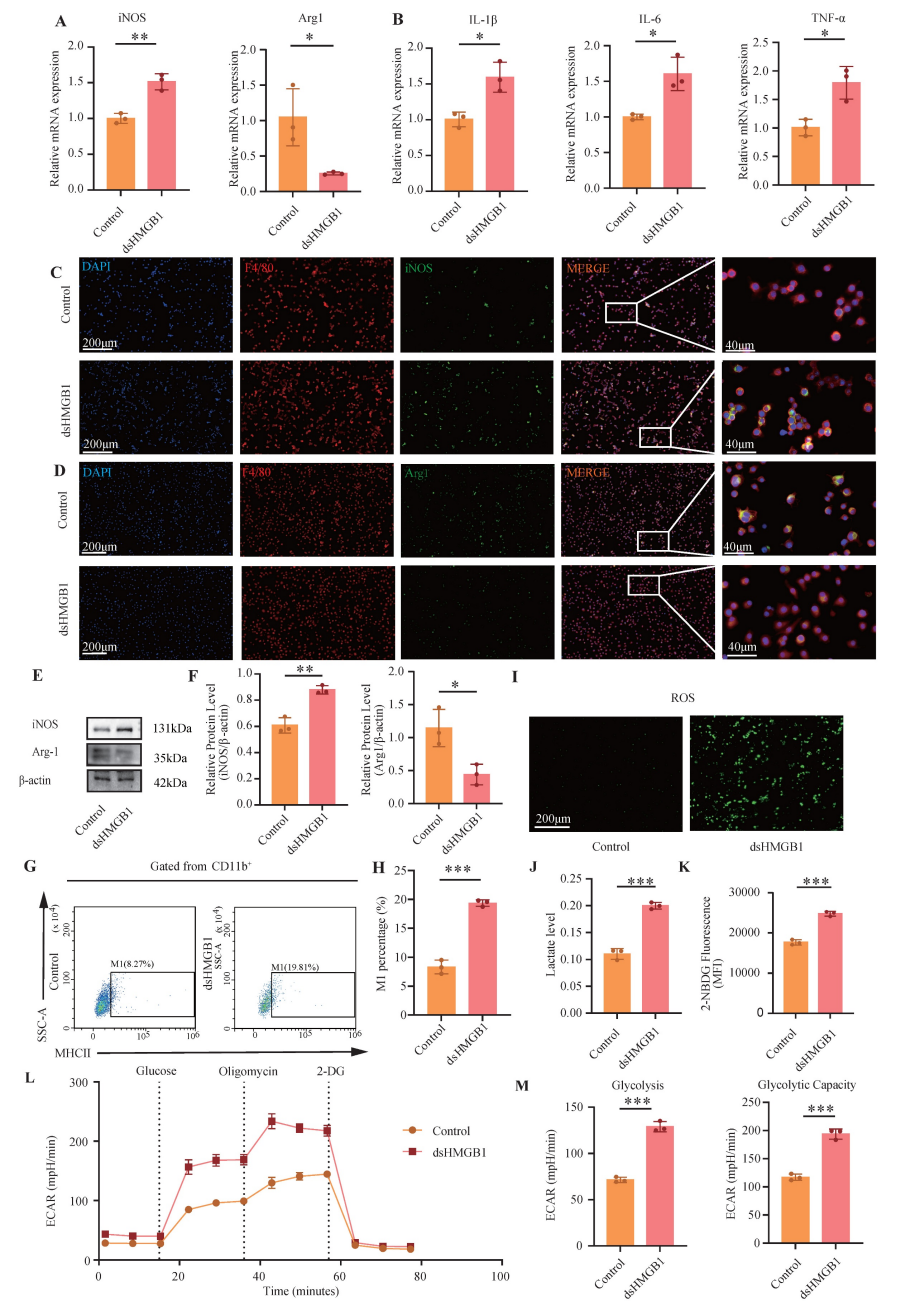
** dsHMGB1 promotes glycolytic-dependent M1 macrophage polarization.** (A) RT-qPCR was used to detect the expression of iNOS and Arg1 at RNA level between control and dsHMGB1 group based on macrophage; (B) The RNA levels of inflammatory factors IL-1β, IL-6 and TNF-α between control and dsHMGB1 groups by RT-qPCR; (C-D) The iNOS and Arg1 between the control and dsHMGB1 groups was detected by immunofluorescence; (E-F) Western blot detected the expression of iNOS, Arg1, the marker of macrophage polarization between the control and dsHMGB1 groups at the protein level; (G-H) Flow cytometry was used to detect the M1 polarization of macrophages between control and dsHMGB1 groups; (I) ROS levels between control and dsHMGB1 groups; (J) Lactate levels between the control and dsHMGB1 groups; (K) 2-NBDG was used to measure glucose uptake between the control and dsHMGB1 groups; (L-M) Real-time monitoring of extracellular acidification rates (ECAR) in iBMDMs following sequential administration of glucose, oligomycin, and 2-deoxyglucose (2-DG). *P < 0.05; **P < 0.01; ***P < 0.001.

First, RNA-level verification was performed. The RT-qPCR results revealed a significant increase in the M1 macrophage polarization marker iNOS and IL-1β, IL-6, and TNF-α in the dsHMGB1 group compared with those in the control group (all *P* < 0.05, **Figure 5A-B**). Conversely, a significant decrease in Arg1 expression was observed in dsHMGB1 group (*P* < 0.05, **Figure 5A**). The IF staining results revealed significant up-regulation of iNOS expression in macrophages following dsHMGB1 stimulation, which exceeded the levels observed in the control group, whereas Arg1 had the opposite effect (**Figure 5C-D**). This finding was further confirmed by western blotting. In comparison to the control group, iNOS expression was significantly greater in the dsHMGB1 group, whereas Arg1 expression was lower in the dsHMGB1 group than this in the control group (all *P* < 0.05, **Figure 5E-F**). Flow cytometry analysis revealed a significantly greater proportion of M1 macrophage in the dsHMGB1 group than this in the control group (*P* < 0.05, **Figure 5G-H**), thereby providing further evidence supporting the role of dsHMGB1 in driving M1 macrophage polarization. In addition, dsHMGB1 also has a certain inhibitory effect on M2 (*P* < 0.05, **Figure S1D-E**).

To further validate the metabolic changes in macrophages following dsHMGB1 stimulation, we conducted assays for ROS, lactic acid detection, and glucose uptake tests. **Figure 5I** shows that the ROS levels in the dsHMGB1 group were significantly higher than those in the control group. The lactic acid assay results revealed a significant increase in lactic acid in the dsHMGB1 group (*P* < 0.05, **Figure 5J**). Furthermore, the glucose uptake capacity in the dsHMGB1 group was markedly higher compared to the control group (*P* < 0.05, **Figure 5K**). Therefore, dsHMGB1 enhances M1 polarization through modulation of glycolytic activity in macrophages. Seahorse XF assays revealed that 24 h of exposure to dsHMGB1 induced a pronounced metabolic shift toward glycolysis, as indicated by a significant increase in the extracellular acidification rate (ECAR). Both glycolysis and glycolytic capacity were markedly elevated following stimulation (**Figure 5L-M**), indicating that dsHMGB1 enhances glycolytic reprogramming in macrophages.

### 3.6 dsHMGB1 activates Jak2/Stat1 transcription to upregulate Pfkp

**Figure 6:**
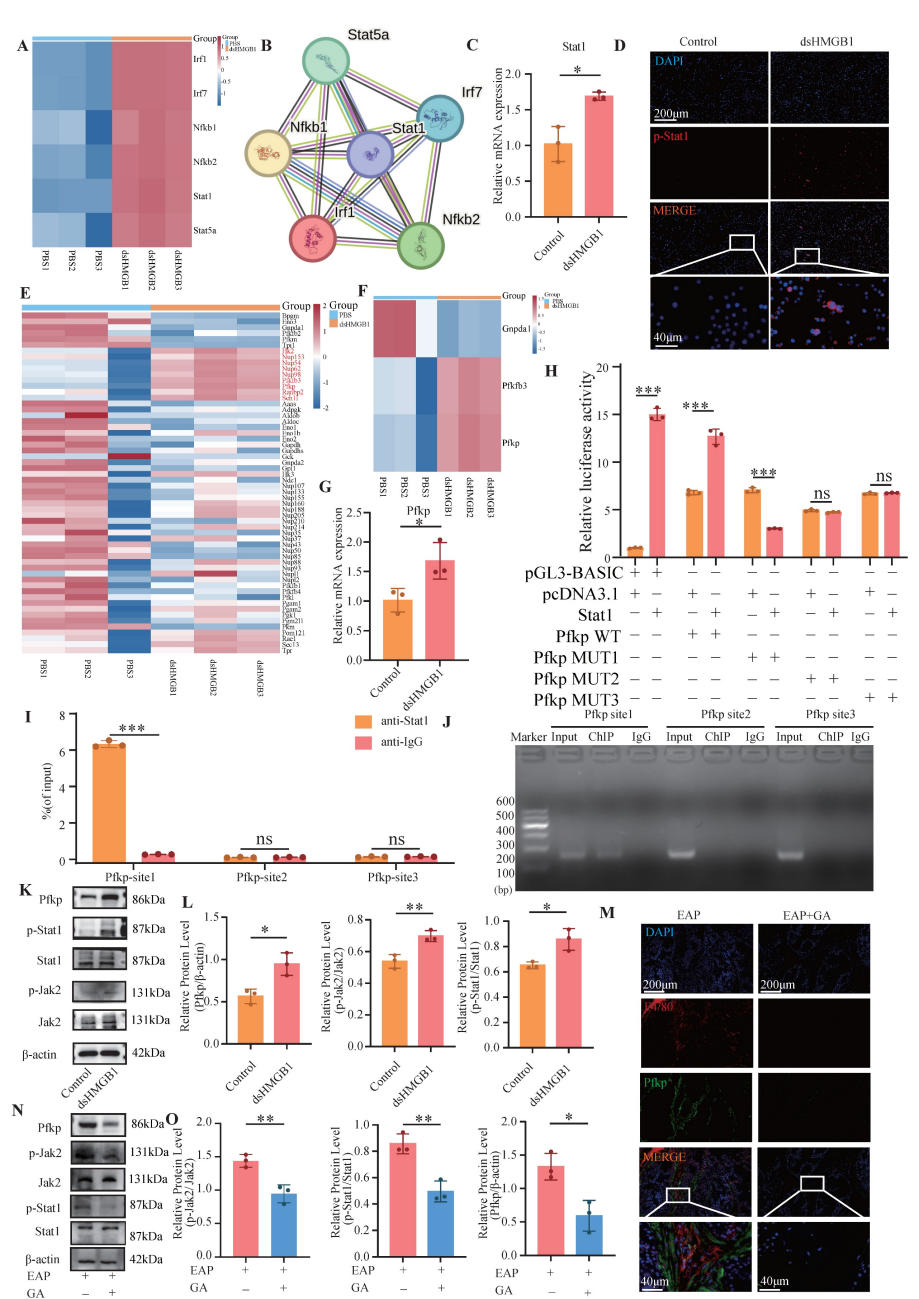
** dsHMGB1 activates Jak2/Stat1 transcription to up-regulate Pfkp.** (A) RNA sequence analysis of TF; (B)Interaction analysis based on STRING; (C) The RNA levels of Stat1 between control and dsHMGB1 groups by RT-qPCR; (D) The IF staining of p-Stat1 between control and dsHMGB1 group; (E-F) RNA sequence analysis of key genes of glycolysis; (G)Differences in the expression of Pfkp at RNA level between the control and dsHMGB1 groups based on macrophage; (H) Dual luciferase detected the binding of TF Stat1 to Pfkp; (I-J) The binding of transcription factor Stat1 to downstream target gene Pfkp was detected by ChIP-qPCR; (K-L) Western blot detected the expression of Jak2, p-Jak2, Stat1, p-Stat1 and Pfkp between the control and dsHMGB1 groups at the protein level; (M) The IF co-staining of F4/80 and Pfkp between the EAP and EAP+GA groups; (N-O) Western blot detected the expression of Jak2, p-Jak2, Stat1, p-Stat1 and Pfkp between the EAP and EAP+GA groups at the protein level. *P < 0.05; **P < 0.01; ***P < 0.001.

To elucidate the specific mechanism underlying dsHMGB1-induced M1 macrophage polarization, we conducted an analysis of the transcriptomic data (GSE200210), identified the intersection between DEGs (*P* < 0.05, |log_2_Fc| > 2) and transcription factor (TF) subsets, and subsequently performed bioinformatics analysis (**Figure 6A**). The analysis of TF interactions using the STRING database revealed that Stat1 occupied a central position, indicating its potential significance in mediating M1 macrophage polarization induced by dsHMGB1 (**Figure 6B**). RT-qPCR analysis showed that Stat1 expression was markedly upregulated in the dsHMGB1 group compared to the control group (**Figure 6C**). The IF results also confirmed that p-Stat1 was highly expressed in the dsHMGB1 group (**Figure 6D**). Furthermore, to identify the key enzymes involved in glycolytic metabolism, we retrieved all glycolysis-associated enzymes and performed an analysis to filter the increased enzymes after dsHMGB1 treatment, remarkably, our findings revealed a significant upregulation in Pfkp, which is recognized as the key rate-limiting enzyme within glycolytic metabolism (**Figure 6E-F**). RT-qPCR analysis demonstrated that Pfkp expression was considerably higher in the dsHMGB1 group compared to the control group (**Figure 6G**). We subsequently knocked down Pfkp using siRNA to verify whether its alteration could directly affect the function of dsHMGB1. The results revealed that after Pfkp was knocked down, the pro-inflammatory ability and M1 polarization level of dsHMGB1 significantly decreased (all *P* <0.05, **Figure S2**).

A literature review revealed that the Jak2/Stat1 signaling pathway is critically involved in the regulation of M1 macrophage polarization^39^. We conducted ChIP-qPCR and dual luciferase experiments to validate the binding sites of Stat1 and Pfkp. The results of the dual-luciferase assay demonstrated the binding of Stat1 to promoter site 1 of the Pfkp, with a subsequent reduction in luciferase activity upon mutation (**Figure 6H**). The ChIP-qPCR results demonstrated robust binding of Stat1 to the promoter site 1 of Pfkp, but not to other sites (**Figure 6I-J**). The protein levels of p-Jak2, p-Stat1, and Pfkp were markedly increased in the dsHMGB1 group when compared to the control group (**Figure 6K-L**). In addition, the levels of p-Jak2, p-Stat1 and Pfkp was significantly decreased in EAP mice after GA treatment (**Figure 6M-O**). Therefore, we suggest that dsHMGB1 transcriptionally regulates Pfkp by activating the Jak2/Stat1 signaling pathway.

### 3.7 Fludarabine and si-Jak2/Stat1 mitigates dsHMGB1-mediated M1 polarization

**Figure 7:**
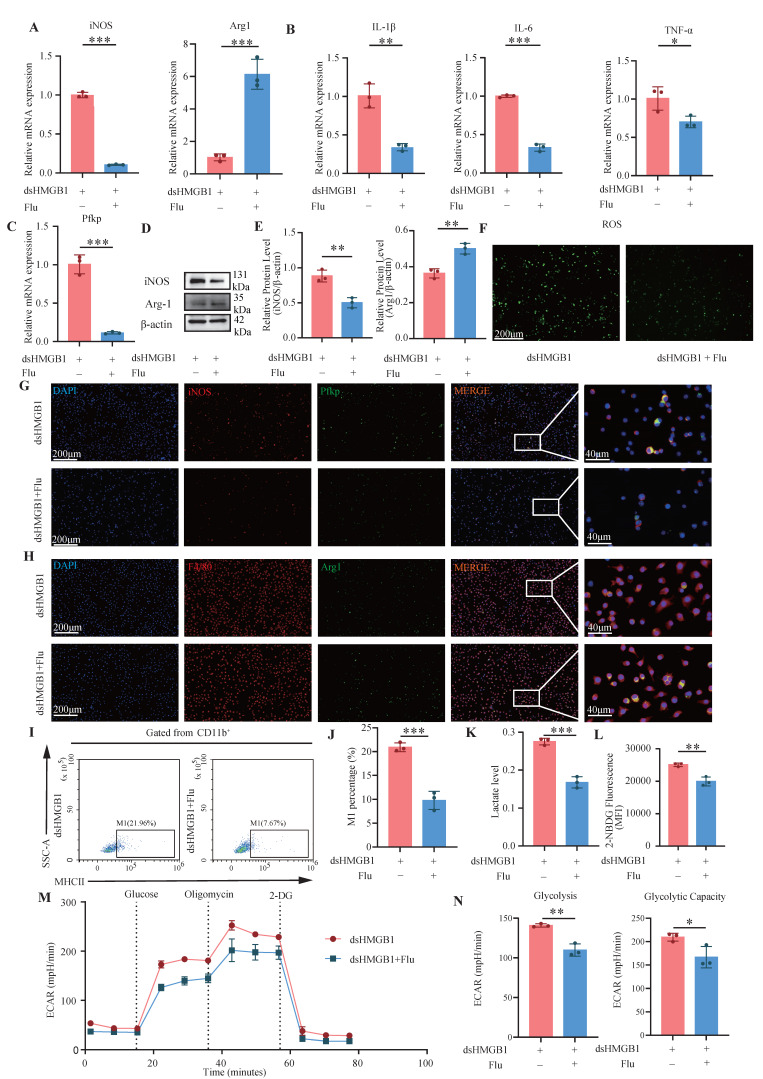
** Fludarabine mitigates dsHMGB1-mediated M1 polarization.** (A) RT-qPCR detect the expression of iNOS and Arg1 at RNA level between dsHMGB1 and dsHMGB1+Flu group based on macrophage; (B-C) The expression of IL-1β, IL-6, TNF-α and Pfkp at RNA level between dsHMGB1 and dsHMGB1+Flu group based on macrophage; (D-E) The protein level of iNOS and Arg1 between the dsHMGB1 and dsHMGB1+Flu groups was detected by western blot. (F) ROS levels between dsHMGB1 and dsHMGB1+Flu groups; (G-H) The iNOS, Pfkp and Arg1 between the dsHMGB1 and dsHMGB1+Flu groups was detected by immunofluorescence; (I-J) Flow cytometry was used to detect the M1 polarization of macrophages between dsHMGB1 and dsHMGB1+Flu groups; (K)Lactate levels between the dsHMGB1 and dsHMGB1+Flu groups; (L) 2-NBDG was used to measure glucose uptake between the dsHMGB1 and dsHMGB1+Flu groups. (M-N) Real-time monitoring of extracellular acidification rates (ECAR) in iBMDMs following sequential administration of glucose, oligomycin, and 2-DG. *P < 0.05; **P < 0.01; ***P < 0.001.

The inhibition of Stat1 activation is significantly mediated by fludarabine^40^. Therefore, we investigated the effect of dsHMGB1 on M1 macrophage polarization by the inhibition of Stat1 with fludarabine. The RT-qPCR results revealed a significantly greater expression of iNOS in the dsHMGB1 group than this in the dsHMGB1+Flu group, whereas the opposite trend was detected for Arg1 (all *P* < 0.05, **Figure 7A**). Similarly, patterns of pro-inflammatory IL-1β, IL-6, and TNF-α expression were consistent with those of iNOS expression (all *P* < 0.05, **Figure 7B**). Fludarabine also inhibited the up-regulation of Pfkp induced by dsHMGB1(*P* < 0.05, **Figure 7C**). Western blotting additionally demonstrated that fludarabine attenuated dsHMGB1-induced M1 macrophage polarization (all *P* < 0.05, **Figure 7D-E**). Fludarabine significantly inhibited the level of ROS stimulated by dsHMGB1 (**Figure 7F**). Additionally, IF staining of iNOS and Pfkp revealed that fludarabine significantly inhibited the upregulation of both proteins induced by dsHMGB1, while Arg1 had the opposite effect (**Figure 7G-H**). Flow cytometry analysis revealed that fludarabine significant inhibited dsHMGB1-induced M1 macrophage polarization (**Figure 7I-J**). In addition, fludarabine also has a certain promoting effect on M2 (*P* < 0.05, **Figure S1F-G**). Moreover, the lactate acid and glucose uptake capacity of the macrophages in the dsHMGB1+Flu group was lower than that in the dsHMGB1 group (**Figure 7K-L**). The Seahorse XF assays indicated that after Stat1 was inhibited with fludarabine, the ECAR decreased, and the glycolysis and capacity were reduced (**Figure 7 M-N**). In addition, we individually knocked down Jak2 and Stat1 using siRNA to further validate their roles in this signaling pathway. The results were consistent with previous findings, showing that the knockdown of these two genes both contributed to the down-regulation of inflammatory factor expression and M1 polarization marker expression (**Figure S3-S4**).

## 4. Discussion

**Figure 8:**
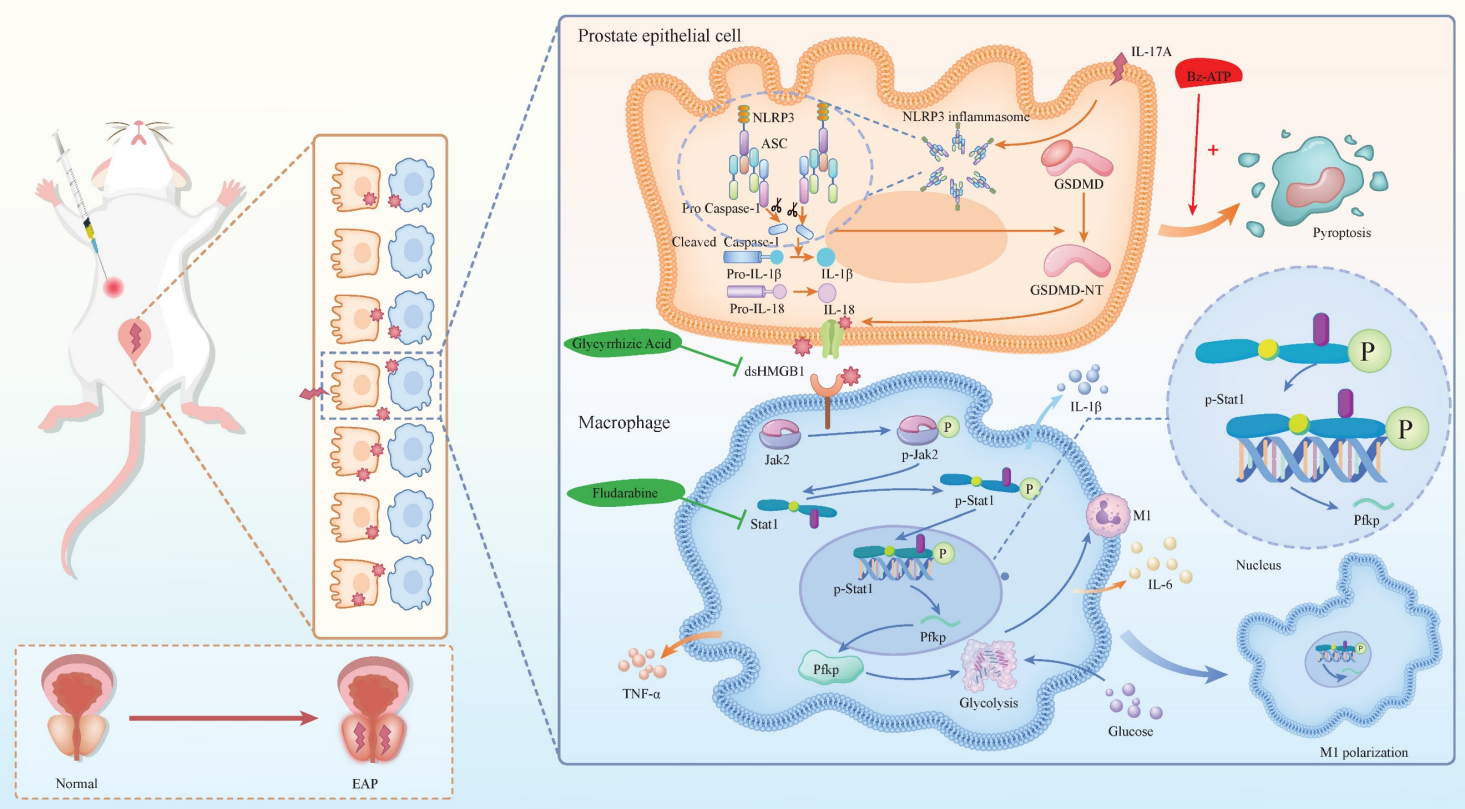
** The mechanism diagram.** The mechanism diagram showed that dsHMGB1 released from IL-17A induced pyrosis of prostatic epithelial cells, which affected Jak2/Stat1 transcriptional regulation of Pfkp, led to changes in macrophage glucose metabolism, and further affected M1 polarization process (By Figdraw).

Chronic prostatitis is characterized by the infiltration of various immune cells, with macrophages playing a pivotal role in the inflammatory process. IL-17A and Bz-ATP have been shown to significantly influence the pyroptosis state of EAP prostate tissue, as well as the release of the dsHMGB1. Studies have indicated that dsHMGB1 plays a crucial role in regulating the M1 polarization state of macrophages. Specifically, the activation of the Jak2/Stat1 pathway is known to be a central regulatory mechanism in the process of M1 macrophage polarization. Under the induction of dsHMGB1, Stat1 regulates the transcription of Pfkp, reprogramming the glycolytic metabolism of macrophages and thereby promoting M1 macrophage polarization. In our investigation of the use of GA and Flu to treat EAP and macrophages, we confirmed the potential therapeutic efficacy of these corresponding targets. In summary, our findings provide novel insights into the pyroptosis mechanism of chronic prostatitis, elucidate the regulatory role of macrophages, and validate the feasibility of targeting these pathways for therapeutic intervention in CP/CPPS (**Figure 8**).

Our research revealed that IL-17A induced pyroptosis in PECs by activating the NLRP3 inflammasome pathway, which subsequently triggered the release of HMGB1—a key damage-associated molecular pattern (DAMP) that amplifies inflammation and tissue injury. IL-17A is an important inflammatory factor that plays a key role in various inflammatory processes. Studies have shown that in an asthma mouse model, the expression level of GSDMD is increased, and the concentration of IL-17A in the serum is also significantly increased^41^. Furthermore, studies have demonstrated that IL-17A mediates pyroptosis of NEC through the ERK pathway^14^. IL-17A has also been shown to induce pyroptosis in IECs, with this process being driven by the cleavage of Caspase-1 and GSDMD. Notably, the use of Caspase-1 inhibitors prevents IL-17A-induced cytotoxicity^42^. In addition, IL-17 promotes the upregulation of NLRP3, Caspase-1 and GSDMD, which lead to damage to osteoblasts, including porosity, swelling, and rupture. Inhibition of NLRP3 can block these effects^43^. Activation of the IL-17 signaling pathway enhances pyroptosis in pneumonia-induced sepsis, and the expression of HMGB1, one of the key molecules in this pathway, is also increased^44^. Notably, HMGB1, an inflammatory mediator, is released through the cell membrane when pyroptosis occurs, and inhibiting pyroptosis can block the release of HMGB1^45^. Our previous research has revealed that the P2X7R-NEK7-NLRP3 axis promotes GSDMD-NT-mediated pyroptosis of prostate epithelial cells and contributes to the onset of chronic prostatitis. As such, Bz-ATP, acting as a P2X7R agonist, mediates pyroptosis via the NLRP3 and GSDMD pathways, increases the proportion of Th17 cells, and thereby enhances IL-17A expression^28^. In this study, we found that the administration of an anti-IL-17A monoclonal antibody alleviated pyroptosis of prostate epithelial cells in EAP mice. On the basis of our previous findings, we subsequently performed a rescue experiment in which EAP mice were first treated with an anti-IL-17A monoclonal antibody followed by Bz-ATP injection. The findings indicated that Bz-ATP reversed the protective effect of the anti-IL-17A antibody on prostate epithelial cell pyroptosis, which was consistent with our earlier observations.

Polarization of macrophages plays an important role in the onset and development of EAP^46^. Previous studies have applied rhHMGB1 or rmHMGB1 to stimulate THP-1 and RAW264.7 macrophages, respectively, resulting in significant upregulation of M1-like related mRNA expression while downregulating M2-like related mRNA expression^47^. Three subtypes of HMGB1, namely, dsHMGB1, frHMGB1, and oxHMGB1, have been described. Previous transcriptome sequencing results revealed that BMDMs stimulated with dsHMGB1 presented a transcriptomic profile that partially overlapped but was clearly distinguishable from those activated by LPS/IFNγ or LPS alone. In contrast, frHMGB1 failed to elicit any notable alterations in gene expression. Among the HMGB1 isoforms, dsHMGB1 is uniquely recognized for its pro-inflammatory activity, which involves triggering cytokine production through the TLR4-MyD88-NFκB signaling cascade. Furthermore, research has shown that frHMGB1 can induce the chemotaxis of BMDMs but does not induce their polarization, whereas dsHMGB1 is capable of promoting their polarization toward the M1 phenotype^24^, ^25^, ^48^. OxHMGB1 does not exhibit chemokine or cytokine activity^49^-^51^. Another study demonstrated that dsHMGB1 alone was sufficient to initiate neuroinflammatory and sickness responses when faced with a pro-inflammatory stimulus. Additionally, dsHMGB1 was shown to directly trigger a pro-inflammatory response in microglia during immune challenges, suggesting that its neuroinflammatory effects may be partly attributed to its direct impact on microglia^52^. Our experiments in iBMDM yielded similar results, showing that the HMGB1 inhibitor GA significantly reduced M1 macrophage polarization and infiltration in prostate tissue following the establishment of an EAP mouse model. Although GA has potential as a therapeutic agent for chronic prostatitis, further verification is needed.

The findings of this study suggest that Stat1 occupies a central position among the transcription factors that are upregulated, suggesting its pivotal role in promoting M1 polarization via dsHMGB1^24^, ^25^. Previous research has emphasized the significance of the Jak2/Stat1 signaling pathway in governing the polarization of macrophages toward the M1 phenotype^53^. In our study, we confirmed the activation of the Jak2/Stat1 pathway by dsHMGB1 in iBMDMs, leading to M1 polarization, which can be effectively suppressed by fludarabine, a Stat1 inhibitor.

The polarization of macrophages is influenced by energy metabolism^54^, ^55^. Studies have shown that METTLL3-156a interacts with lactate dehydrogenase A to increase macrophage glycolysis, thereby promoting M1 polarization^56^. In our previous research, we reported that during the LPS stimulation, knockdown of CXCR4 expression would reduce the expression level of c-MYC, which in turn decreased the levels of Pfkfb3, a key enzyme in glucose metabolism. This ultimately inhibited glycolytic metabolism and promoted the transformation of macrophages from the M1 to the M2^26^. As noted above, both previous studies and our current findings demonstrated that dsHMGB1 can induce M1 macrophage polarization. However, it remains unclear whether dsHMGB1 can induce M1 polarization via the glycolytic metabolic pathway. In the present study, Pfkp served as a crucial rate-limiting enzyme in regulating glycolysis. We observed significant upregulation of Pfkp in M1 macrophages polarized in response to dsHMGB1 stimulation, resulting in increased glycolytic activity. In addition, luciferase and ChIP-qPCR assays provided further evidence that Stat1 functions as a transcriptional activator of Pfkp. Therefore, we innovatively demonstrated that dsHMGB1 induced M1 macrophage polarization through Jak2/Stat1 mediated transcriptional regulation of the key glycolytic enzyme Pfkp, thereby promoting glycolytic metabolism in the EAP model.

There are several limitations in our research. First, although GA and Flu showed efficacy in alleviating EAP symptoms, their long-term safety, pharmacokinetics and off-target effects remain uncharacterized. Second, this study utilized a NOD mouse model of EAP and iBMDM. Although these models can effectively simulate the key features of human chronic prostatitis, species differences in the prostate microenvironment may affect the translation of mechanisms to the clinic. Future validation of the core findings in organoids or humanized models is needed.

## 5. Conclusion

Our research demonstrated that dsHMGB1, which is released from IL-17A-induced pyroptotic prostate epithelial cells, plays a pivotal role in driving M1 polarization in EAP model. This process occurs through the activation of the Jak2/Stat1 signaling pathway, which regulates the transcription of the key glycolytic enzyme Pfkp. Targeting dsHMGB1 or Stat1 may help control M1 polarization and inflammatory cytokine production, suggesting potential therapeutic strategies for managing CP/CPPS in the future.

## Supplementary Material

Supplementary figures and tables.

## Abbreviations

CP/CPPS: chronic prostatitis/chronic pelvic pain syndrome
NIH: National Institute of Health
WBC: white blood cells
LUTS: lower urinary tract symptoms
PAgs: prostate-associated antigens
Th17: T helper 17
IL-17: interleukin 17
GSDMD: gasdermin D
NEC: nasal epithelial cells
xuanbi yuyang decoction: XBD
IECs: intestinal epithelial cells
PECs: prostate epithelial cells
HMGB1: high-mobility group box-1
CCECs: canine corneal epithelial cells
dsHMGB1: disulfide HMGB1
frHMGB1: fully reduced HMGB1
oxHMGB1: sulfonyl HMGB1
MIF: migration inhibitory factor
EAP: experimental autoimmune prostatitis
SPF: specific pathogen-free
SD: Sprague-Dawley
CFA: complete Freund's adjuvant
GA: glycyrrhizic acid
iBMDM: immortalized bone marrow derived macrophages
FBS: fetal bovine serum
Flu: fludarabine
siRNA: small interfering RNA
H&E: hematoxylin-Eosin
IF: immunofluorescence
IHC: immunohistochemical
ROS: reactive oxygen species
FCM: flow cytometry
RT-qPCR: real-time quantitative polymerase chain reaction
DEG: differential expressed gene
ChIP-qPCR: chromatin Immunoprecipitation qPCR
2-DG: 2-deoxyglucose
TF: transcription factor
DAMP: damage-associated molecular patter

## References

[1] Murphy SF, Schaeffer AJ, Thumbikat P (2014). Immune mediators of chronic pelvic pain syndrome. Nat Rev Urol.

[2] Krieger JN, Nyberg L Jr, Nickel JC (1999). NIH consensus definition and classification of prostatitis. JAMA.

[3] McNaughton Collins M, Pontari MA, O'Leary MP, Calhoun EA, Santanna J, Landis JR (2001). Quality of life is impaired in men with chronic prostatitis: the Chronic Prostatitis Collaborative Research Network. J Gen Intern Med.

[4] Wazir J, Ullah R, Li S, Hossain MA, Diallo MT, Khan FU (2019). Efficacy of acupuncture in the treatment of chronic prostatitis-chronic pelvic pain syndrome: a review of the literature. Int Urol Nephrol.

[5] Clemens JQ, Mullins C, Ackerman AL, Bavendam T, van Bokhoven A, Ellingson BM (2019). Urologic chronic pelvic pain syndrome: insights from the MAPP Research Network. Nat Rev Urol.

[6] Hua X, Ge S, Zhang M, Mo F, Zhang L, Zhang J (2021). Pathogenic Roles of CXCL10 in Experimental Autoimmune Prostatitis by Modulating Macrophage Chemotaxis and Cytokine Secretion. Front Immunol.

[7] Chen J, Meng J, Li X, Li X, Liu Y, Jin C (2022). HA/CD44 Regulates the T Helper 1 Cells Differentiation by Activating Annexin A1/Akt/mTOR Signaling to Drive the Pathogenesis of EAP. Front Immunol.

[8] Motrich RD, Breser ML, Molina RI, Tissera A, Olmedo JJ, Rivero VE (2020). Patients with chronic prostatitis/chronic pelvic pain syndrome show T helper type 1 (Th1) and Th17 self-reactive immune responses specific to prostate and seminal antigens and diminished semen quality. BJU Int.

[9] Du HX, Yue SY, Niu D, Liu C, Zhang LG, Chen J (2022). Gut Microflora Modulates Th17/Treg Cell Differentiation in Experimental Autoimmune Prostatitis via the Short-Chain Fatty Acid Propionate. Front Immunol.

[10] Zhang C, Chen J, Wang H, Chen J, Zheng MJ, Chen XG (2022). IL-17 exacerbates experimental autoimmune prostatitis via CXCL1/CXCL2-mediated neutrophil infiltration. Andrologia.

[11] Murphy SF, Schaeffer AJ, Done J, Wong L, Bell-Cohn A, Roman K (2015). IL17 Mediates Pelvic Pain in Experimental Autoimmune Prostatitis (EAP). PLoS One.

[12] Kesavardhana S, Kanneganti TD (2017). Mechanisms governing inflammasome activation, assembly and pyroptosis induction. Int Immunol.

[13] Al Mamun A, Mimi AA, Zaeem M, Wu Y, Monalisa I, Akter A (2021). Role of pyroptosis in diabetic retinopathy and its therapeutic implications. Eur J Pharmacol.

[14] Li Y, Chang LH, Huang WQ, Bao HW, Li X, Chen XH (2022). IL-17A mediates pyroptosis via the ERK pathway and contributes to steroid resistance in CRSwNP. J Allergy Clin Immunol.

[15] Huang X, Li L, Zheng C, Li J, Chen G, Chen Y (2024). Xuanbi Yuyang Decoction Ameliorates DSS-Induced Colitis by Inhibiting Pyroptosis via Blocking of IL-17 Pathway Activation. J Inflamm Res.

[16] Li Y, Wu Q (2024). KRT6A Inhibits IL-1β-Mediated Pyroptosis of Keratinocytes via Blocking IL-17 Signaling. Crit Rev Eukaryot Gene Expr.

[17] Gaskell H, Ge X, Nieto N (2018). High-Mobility Group Box-1 and Liver Disease. Hepatology communications.

[18] Yang Y, Li S, Liu K, Zhang Y, Zhu F, Ben T (2024). Lipocalin-2-mediated intestinal epithelial cells pyroptosis via NF-κB/NLRP3/GSDMD signaling axis adversely affects inflammation in colitis. Biochim Biophys Acta Mol Basis Dis.

[19] Wang Z, Guo L, Yuan C, Zhu C, Li J, Zhong H (2024). Staphylococcus pseudintermedius induces pyroptosis of canine corneal epithelial cells by activating the ROS-NLRP3 signalling pathway. Virulence.

[20] Tan G, Huang C, Chen J, Zhi F (2020). HMGB1 released from GSDME-mediated pyroptotic epithelial cells participates in the tumorigenesis of colitis-associated colorectal cancer through the ERK1/2 pathway. J Hematol Oncol.

[21] Tang D, Kang R, Zeh HJ, Lotze MT (2023). The multifunctional protein HMGB1: 50 years of discovery. Nat Rev Immunol.

[22] Urbonaviciute V, Fürnrohr BG, Meister S, Munoz L, Heyder P, De Marchis F (2008). Induction of inflammatory and immune responses by HMGB1-nucleosome complexes: implications for the pathogenesis of SLE. The Journal of experimental medicine.

[23] Xue J, Suarez JS, Minaai M, Li S, Gaudino G, Pass HI (2021). HMGB1 as a therapeutic target in disease. J Cell Physiol.

[24] Qu H, Heinbäck R, Salo H, Ewing E, Espinosa A, Aulin C (2022). Transcriptomic Profiling Reveals That HMGB1 Induces Macrophage Polarization Different from Classical M1. Biomolecules.

[25] Yang H, Wang H, Ju Z, Ragab AA, Lundbäck P, Long W (2015). MD-2 is required for disulfide HMGB1-dependent TLR4 signaling. J Exp Med.

[26] Zhang Y, Zhang C, Feng R, Meng T, Peng W, Song J (2024). CXCR4 regulates macrophage M1 polarization by altering glycolysis to promote prostate fibrosis. Cell communication and signaling: CCS.

[27] Zhang F, Meng T, Feng R, Jin C, Zhang S, Meng J (2024). MIF aggravates experimental autoimmune prostatitis through activation of the NLRP3 inflammasome via the PI3K/AKT pathway. Int Immunopharmacol.

[28] Chen L, Liu Y, Yue S, Wang H, Chen J, Ma W (2024). P2X7R Modulates NEK7-NLRP3 Interaction to Exacerbate Experimental Autoimmune Prostatitis via GSDMD-mediated Prostate Epithelial Cell Pyroptosis. Int J Biol Sci.

[29] Niu D, Yue SY, Wang X, Li WY, Zhang L, Du HX (2024). High glucose intake exacerbates experimental autoimmune prostatitis through mitochondrial reactive oxygen species-dependent TGF-β activation-mediated Th17 differentiation. Int Immunopharmacol.

[30] Rabadi M, Kim M, Li H, Han SJ, Choi Y, D'Agati V (2018). ATP induces PAD4 in renal proximal tubule cells via P2X7 receptor activation to exacerbate ischemic AKI. Am J Physiol Renal Physiol.

[31] Hua S, Ma M, Fei X, Zhang Y, Gong F, Fang M (2019). Glycyrrhizin attenuates hepatic ischemia-reperfusion injury by suppressing HMGB1-dependent GSDMD-mediated kupffer cells pyroptosis. Int Immunopharmacol.

[32] Yang G, Zhang Q, Tan J, Xiong Y, Liang Y, Yan J (2023). HMGB1 induces macrophage pyroptosis in chronic endometritis. Int Immunopharmacol.

[33] Ma J, Chen T, Wu S, Yang C, Bai M, Shu K (2019). iProX: an integrated proteome resource. Nucleic Acids Res.

[34] Chen T, Ma J, Liu Y, Chen Z, Xiao N, Lu Y (2022). iProX in 2021: connecting proteomics data sharing with big data. Nucleic Acids Res.

[35] Breser ML, Motrich RD, Sanchez LR, Rivero VE (2017). Chronic Pelvic Pain Development and Prostate Inflammation in Strains of Mice With Different Susceptibility to Experimental Autoimmune Prostatitis. Prostate.

[36] Motrich RD, Maccioni M, Ponce AA, Gatti GA, Oberti JP, Rivero VE (2006). Pathogenic consequences in semen quality of an autoimmune response against the prostate gland: from animal models to human disease. J Immunol.

[37] Akutagawa K, Fujita T, Ouhara K, Takemura T, Tari M, Kajiya M (2019). Glycyrrhizic acid suppresses inflammation and reduces the increased glucose levels induced by the combination of Porphyromonas gulae and ligature placement in diabetic model mice. Int Immunopharmacol.

[38] Kim SW, Jin Y, Shin JH, Kim ID, Lee HK, Park S (2012). Glycyrrhizic acid affords robust neuroprotection in the postischemic brain via anti-inflammatory effect by inhibiting HMGB1 phosphorylation and secretion. Neurobiol Dis.

[39] Chu W, Li YL, Li JJ, Lin J, Li M, Wang J (2023). Guiqi Baizhu prescription ameliorates cytarabine-induced intestinal mucositis by targeting JAK2 to inhibit M1 macrophage polarization. Biomed Pharmacother.

[40] Zhang J, Xu Y, Wei C, Yin Z, Pan W, Zhao M (2023). Macrophage neogenin deficiency exacerbates myocardial remodeling and inflammation after acute myocardial infarction through JAK1-STAT1 signaling. Cell Mol Life Sci.

[41] Wu J, Wang P, Xie X, Yang X, Tang S, Zhao J (2024). Gasdermin D silencing alleviates airway inflammation and remodeling in an ovalbumin-induced asthmatic mouse model. Cell Death Dis.

[42] Lee C, Song JH, Cha YE, Chang DK, Kim YH, Hong SN (2022). Intestinal Epithelial Responses to IL-17 in Adult Stem Cell-derived Human Intestinal Organoids. J Crohns Colitis.

[43] Lei L, Sun J, Han J, Jiang X, Wang Z, Chen L (2021). Interleukin-17 induces pyroptosis in osteoblasts through the NLRP3 inflammasome pathway in vitro. Int Immunopharmacol.

[44] Li LL, Dai B, Sun YH, Zhang TT (2020). The activation of IL-17 signaling pathway promotes pyroptosis in pneumonia-induced sepsis. Ann Transl Med.

[45] Jiao P, Wang Y, Ren G, Chu D, Li Y, Yang Y (2024). Urolithin A exerts a protective effect on lipopolysaccharide-induced acute lung injury by regulating HMGB1-mediated MAPK and NF-κB signaling pathways. Naunyn Schmiedebergs Arch Pharmacol.

[46] Hua X, Zhang J, Ge S, Liu H, Du H, Niu Q (2022). CXCR3 antagonist AMG487 ameliorates experimental autoimmune prostatitis by diminishing Th1 cell differentiation and inhibiting macrophage M1 phenotypic activation. Prostate.

[47] Li Z, Fu WJ, Chen XQ, Wang S, Deng RS, Tang XP (2022). Autophagy-based unconventional secretion of HMGB1 in glioblastoma promotes chemosensitivity to temozolomide through macrophage M1-like polarization. J Exp Clin Cancer Res.

[48] Salo H, Qu H, Mitsiou D, Aucott H, Han J, Zhang XM (2021). Disulfide and Fully Reduced HMGB1 Induce Different Macrophage Polarization and Migration Patterns. Biomolecules.

[49] Tang Y, Zhao X, Antoine D, Xiao X, Wang H, Andersson U (2016). Regulation of Posttranslational Modifications of HMGB1 During Immune Responses. Antioxid Redox Signal.

[50] Raucci A, Di Maggio S, Scavello F, D'Ambrosio A, Bianchi ME, Capogrossi MC (2019). The Janus face of HMGB1 in heart disease: a necessary update. Cell Mol Life Sci.

[51] Chen R, Kang R, Tang D (2022). The mechanism of HMGB1 secretion and release. Exp Mol Med.

[52] Frank MG, Weber MD, Fonken LK, Hershman SA, Watkins LR, Maier SF (2016). The redox state of the alarmin HMGB1 is a pivotal factor in neuroinflammatory and microglial priming: A role for the NLRP3 inflammasome. Brain Behav Immun.

[53] Cheng Y, Li J, Wang L, Wu X, Li Y, Xu M (2023). Eriocalyxin B ameliorated Crohn's disease-like colitis by restricting M1 macrophage polarization through JAK2/STAT1 signalling. Eur J Pharmacol.

[54] Chen R, Wang J, Dai X, Wu S, Huang Q, Jiang L (2022). Augmented PFKFB3-mediated glycolysis by interferon-γ promotes inflammatory M1 polarization through the JAK2/STAT1 pathway in local vascular inflammation in Takayasu arteritis. Arthritis Res Ther.

[55] Chen S, Li S, Wang H (2024). Remodeling tumor-associated macrophages in the tumor microenvironment. Oncol Transl Med.

[56] Xie L, Deng X, Li X, Li X, Wang X, Yan H (2024). CircMETTL3-156aa reshapes the glycolytic metabolism of macrophages to promote M1 polarization and induce cytokine storms in sHLH. Cell Death Discov.

