# Supplementary material for: dsHMGB1, released from IL-17A-induced pyroptotic prostate epithelial cells, drives M1 polarization by promoting Pfkp-mediated glycolysis via Jak2/Stat1 transcription in experimental autoimmune prostatitis
Source: Int J Biol Sci. 2025 Sep 3;21(13):5725–43. doi: 10.7150/ijbs.113908 (PMC12509684; doi:10.7150/ijbs.113908)
Supplement: Supplementary file 1 — Supplementary figures and tables. [file ijbsv21p5725s1.pdf]

Table S1. Primers sequences for siRNA

Gene	Sense	Antisense
Jak2	GGAACAUAUUGGUGGAAAATT	UUUUCCACCAAUAUGUUCCTT
Stat1	AGAAGGAGCUGGACAGUAATT	UUACUGUCCAGCUCCUUCUTT
Pfkp	CGUGCACUUGACAGAGAAATT	UUUCUCUGUCAAGUGCACGTT
Negative Control	UUCUCCGAACGUGUCACGUTT	ACGUGACACGUUCGGAGAATT

Table S2. Antibodies used in Western blot (WB), IHC and IF

Name	Vendor	Cat#	Dilution ratio
β-actin	Affinity	AF7018	1:1000 (WB)
PCK	MCE	HY-P81164	1:200 (IF)
α-SMA	Affinity	AF1032	1:200 (IF)
CD4	Immunoway	YT0762	1:200 (IF)
F4/80	Affinity	DF2789	1:200 (IF)
HMGB1	Affinity	AF7020	1:1000(WB); 1:200 (IHC, IF)
IL-18	Affinity	DF6252	1:1000 (WB)
GSDMD	Affinity	AF4012	1:1000 (WB)/1:200(IF)
Caspase1	Affinity	AF5418	1:1000 (WB)/1:200(IF)
Jak2	Immunoway	YT2426	1:1000 (WB)
p-Jak2	Immunoway	YP0155	1:1000 (WB)
Stat1	Cell Signaling Techology	9172T	1:1000 (WB)
p-Stat1	Immunoway	YP0249	1:1000 (WB)/1:200(IF)
Pfkp	Proteintech	13389-1-AP	1:1000 (WB)/1:200(IF)
iNOS	Affinity	AF0199	1:1000 (WB)/1:200(IF)
Arg 1	Affinity	DF6657	1:1000 (WB)/1:200(IF)
NLRP3	Affinity	DF7438	1:1000 (WB)
ASC	Affinity	DF6304	1:1000 (WB)

Table S3. Primers used in RT-qPCR analysis

Gene	Forward primer sequence	Reverse primer sequence
β-actin	GGCTGTATTCCCCTCCATCG	CCAGTTGGTAACAATGCCATGT
IL-1β	TGGGAAACAACAGTGGTCAGG	CCATCAGAGGCAAGGAGGAA
IL-6	TAGTCCTTCCTACCCCAATTTCC	TTGGTCCTTAGCCACTCCTTC
TNF-α	GAGTGACAAGCCTGTAGCC	CTCCTGGTATGAGATAGCAAA
iNOS	CACCAAGCTGAACTTGAGCG	CGTGGCTTTGGGCTCCTC
Arg-1	CCAGAAGAATGGAAGAGTCAGTGT	GCAGATATGCAGGGAGTCACC
HMGB1	GCTGACAAGGCTCGTTATGAA	CCTTTGATTTTGGGGCGGTA
Stat1	TCACAGTGGTTCGAGCTTCAG	CGAGACATCATAGGCAGCGTG
Pfkp	CGCCTATCCGAAGTACCTGGA	CCCCGTGTAGATTCCCATGC

Table S4. Primers sequences for ChIP-qPCR

Gene	Foward	Reverse
Pfkp site1	CAAAGTGAGTTCCAGGACAGC	TTGGGGGTTTTGCTTTGTTA
Pfkp site2	AGTCTGACACCCCTCCTTCC	TGATGAGCACTAAACTGGAGAA
Pfkp site3	TCAGATGTTCTCCAGTTTAGTGC	TCCTGAACTCCCATGATCAAC

Figure S1. Flow cytometry gating strategy and polarization status of M2-type macrophages.

(A) Flow cytometry gating strategy for M1 polarization of splenic macrophages in EAP mice;

(B) Flow cytometry gating strategy for M1 polarization of macrophages in iBMDM;

(C) Flow cytometry gating strategy for M2 polarization of macrophages in iBMDM;

(D-G) The effects of dsHMGB1 and Flu on M2 polarization of iBMDM. *$P < 0.05$; **$P < 0.01$; ***$P < 0.001$.

Figure S2. si-Pfkp inhibits dsHMGB1-induced M1 macrophage polarization.

(A-C) The expression of Pfkp in the si-NC group and the si-Pfkp group was detected by RT-qPCR and Western blot. (D) The expression of IL-1β, IL-6, TNF-α, iNOS and Arg1 at RNA level between si-NC+dsHMGB1 and si-Pfkp +dsHMGB1 groups based on macrophage; (E-F) The protein level of iNOS and Arg1 between the si-NC+dsHMGB1 and si-Pfkp +dsHMGB1 groups was detected by western blot; (G-H) Flow cytometry was used to detect the M1 polarization of macrophages between si-NC+dsHMGB1 and si-Pfkp+dsHMGB1 groups; (I)Lactate levels between the si-NC+dsHMGB1 and si-Pfkp +dsHMGB1 groups; (J) 2-NBDG was used to measure glucose uptake between the si-NC+dsHMGB1 and si-Pfkp +dsHMGB1 groups. *$P < 0.05$; **$P < 0.01$; ***$P < 0.001$.

Figure S3. si-Jak2 inhibits dsHMGB1-induced M1 macrophage polarization.

(A-C) The expression of Jak2 in the si-NC group and the si-Jak2 group was detected by RT-qPCR and Western blot. (D) The expression of IL-1β, IL-6, TNF-α, iNOS and Arg1 at RNA

level between si-NC+dsHMGB1 and si-Jak2+dsHMGB1 groups based on macrophage; (E-F) The protein level of p-Stat1, Stat1, Pfkp, iNOS and Arg1 between the si-NC+dsHMGB1 and si-Jak2+dsHMGB1 groups was detected by western blot; (G-H) Flow cytometry was used to detect the M1 polarization of macrophages between si-NC+dsHMGB1 and si-Jak2+dsHMGB1 groups; (I)Lactate levels between the si-NC+dsHMGB1 and si-Jak2+dsHMGB1 groups; (J) 2-NBDG was used to measure glucose uptake between the si-NC+dsHMGB1 and si-Jak2+dsHMGB1 groups.*$P < 0.05$; **$P < 0.01$; ***$P < 0.001$.

Figure S4. si-Stat1 inhibits dsHMGB1-induced M1 macrophage polarization.

(A-C) The expression of Stat1 in the si-NC group and the si-Stat1 group was detected by RT-qPCR and Western blot; (D) The expression of IL-1β, IL-6, TNF-α, iNOS and Arg1 at RNA level between si-NC+dsHMGB1 and si-Stat1+dsHMGB1 groups based on macrophage; (E-F) The protein level of Pfkp, iNOS and Arg1 between the si-NC+dsHMGB1 and si-Stat1+dsHMGB1 groups was detected by western blot; (G-H) Flow cytometry was used to detect the M1 polarization of macrophages between si-NC+dsHMGB1 and si-Stat1+dsHMGB1 groups; (I)Lactate levels between the si-NC+dsHMGB1 and si-Stat1+dsHMGB1 groups; (J) 2-NBDG was used to measure glucose uptake between the si-NC+dsHMGB1 and si-Stat1+dsHMGB1 groups.*$P < 0.05$; **$P < 0.01$; ***$P < 0.001$.

Figure S1

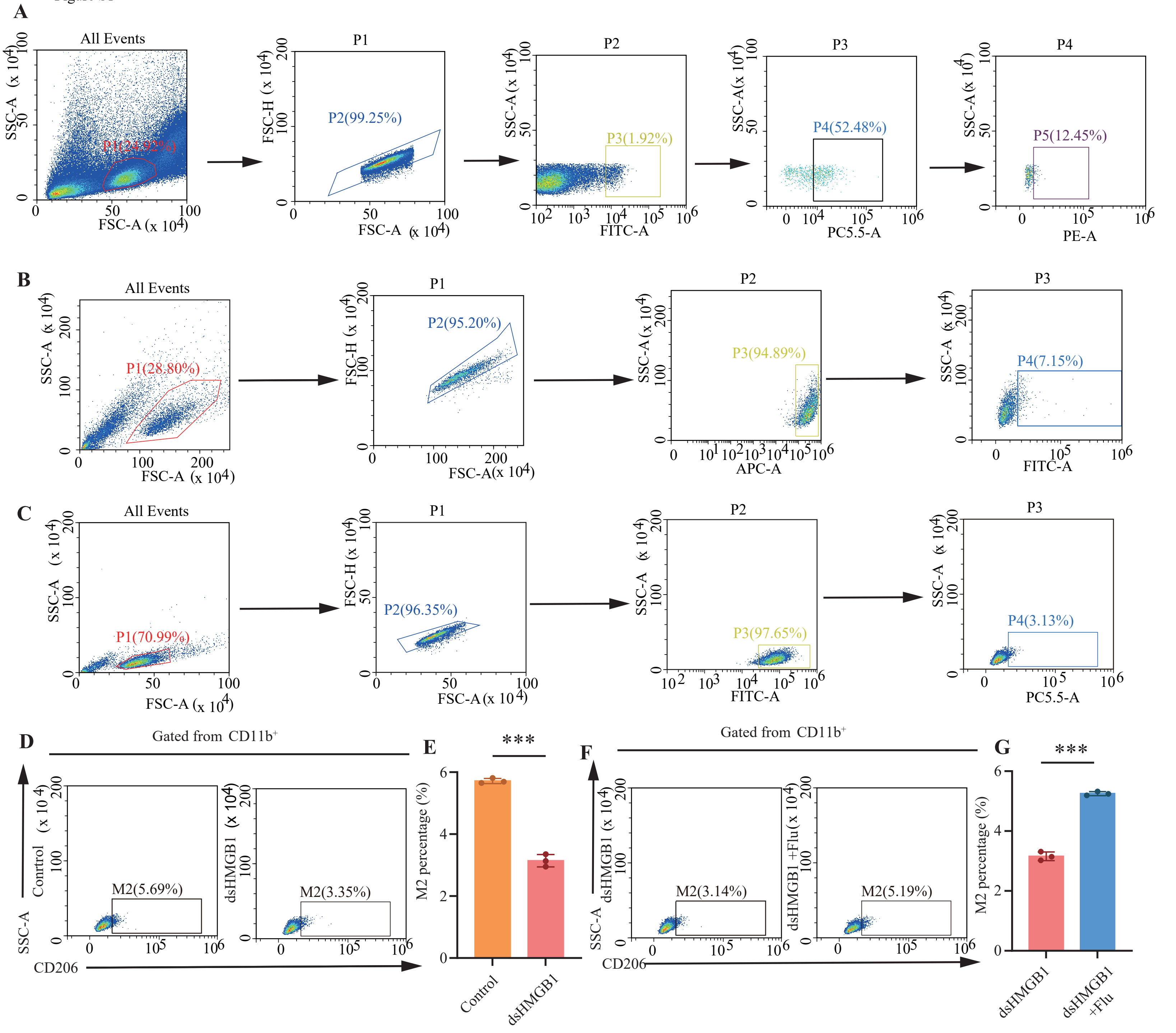
A
All Events
P1(24.92%)
SSC-A (x 10^4)
FSC-A (x 10^4)
P1
P2(99.25%)
FSC-H (x 10^4)
P2
P3(1.92%)
FITC-A
P3
P4(52.48%)
PC5.5-A
P4
P5(12.45%)
PE-A
B
All Events
P1(28.80%)
P2(95.20%)
P3(94.89%)
APC-A
P4(7.15%)
C
All Events
P1(70.99%)
P2(96.35%)
P3(97.65%)
P4(3.13%)
D
Gated from CD11b+
Control
dsHMGB1
M2(5.69%)
M2(3.35%)
CD206
E
***
M2 percentage (%)
Control
dsHMGB1
F
Gated from CD11b+
dsHMGB1+Flu
M2(3.14%)
M2(5.19%)
G
***
dsHMGB1
dsHMGB1 +Flu

Figure S2

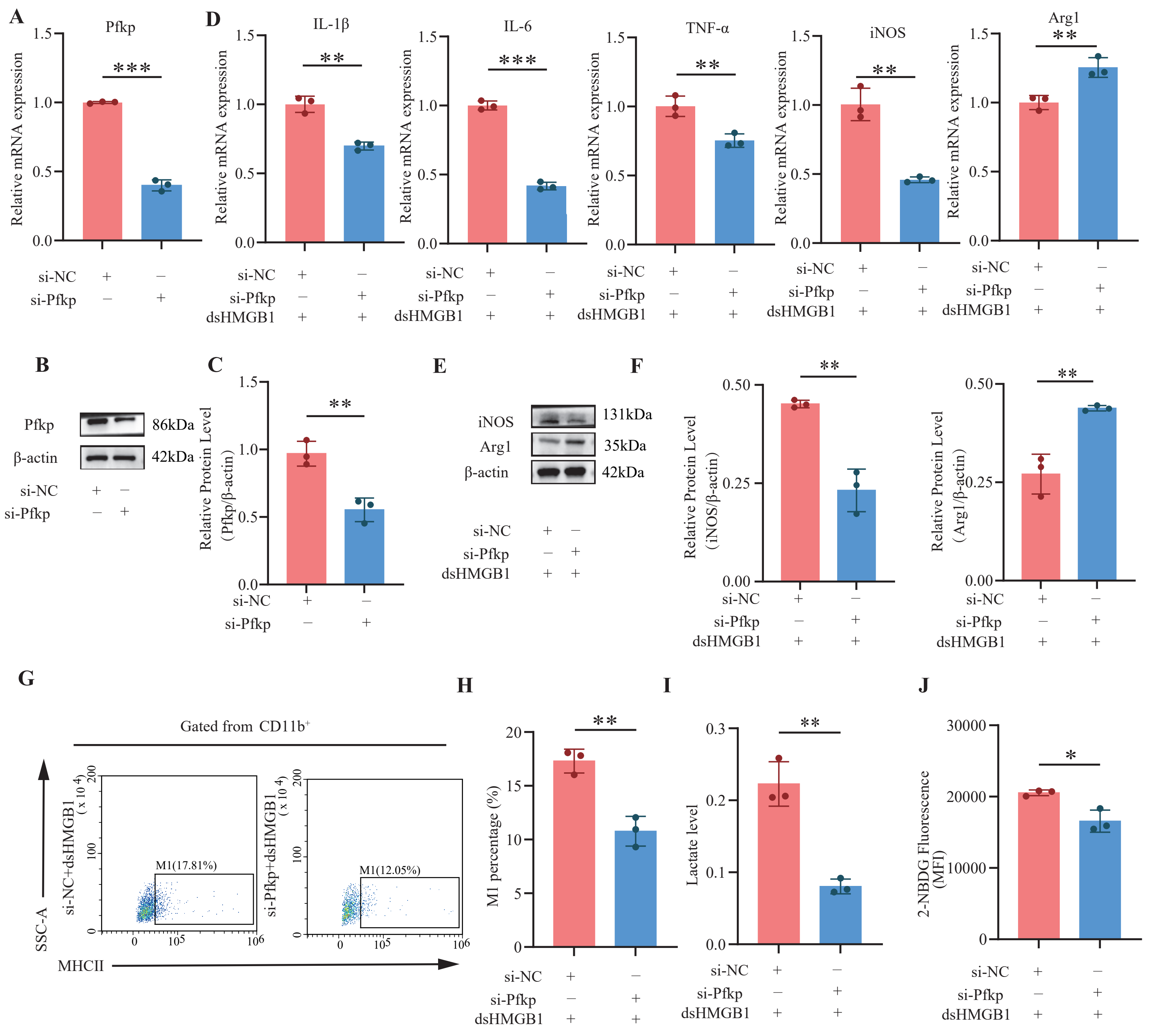

A
Pfkp
Relative mRNA expression
si-NC
si-Pfkp
D
IL-1β
IL-6
TNF-α
iNOS
Arg1
dsHMGB1
B
Pfkp
β-actin
86kDa
42kDa
C
Relative Protein Level (Pfkp/β-actin)
E
iNOS
Arg1
131kDa
35kDa
F
Relative Protein Level (iNOS/β-actin)
Relative Protein Level (Arg1/β-actin)
G
Gated from CD11b+
si-NC+dsHMGB1
si-Pfkp+dsHMGB1
M1(17.81%)
M1(12.05%)
SSC-A
MHCII
H
M1 percentage (%)
I
Lactate level
J
2-NBDG Fluorescence (MFI)

Figure S3

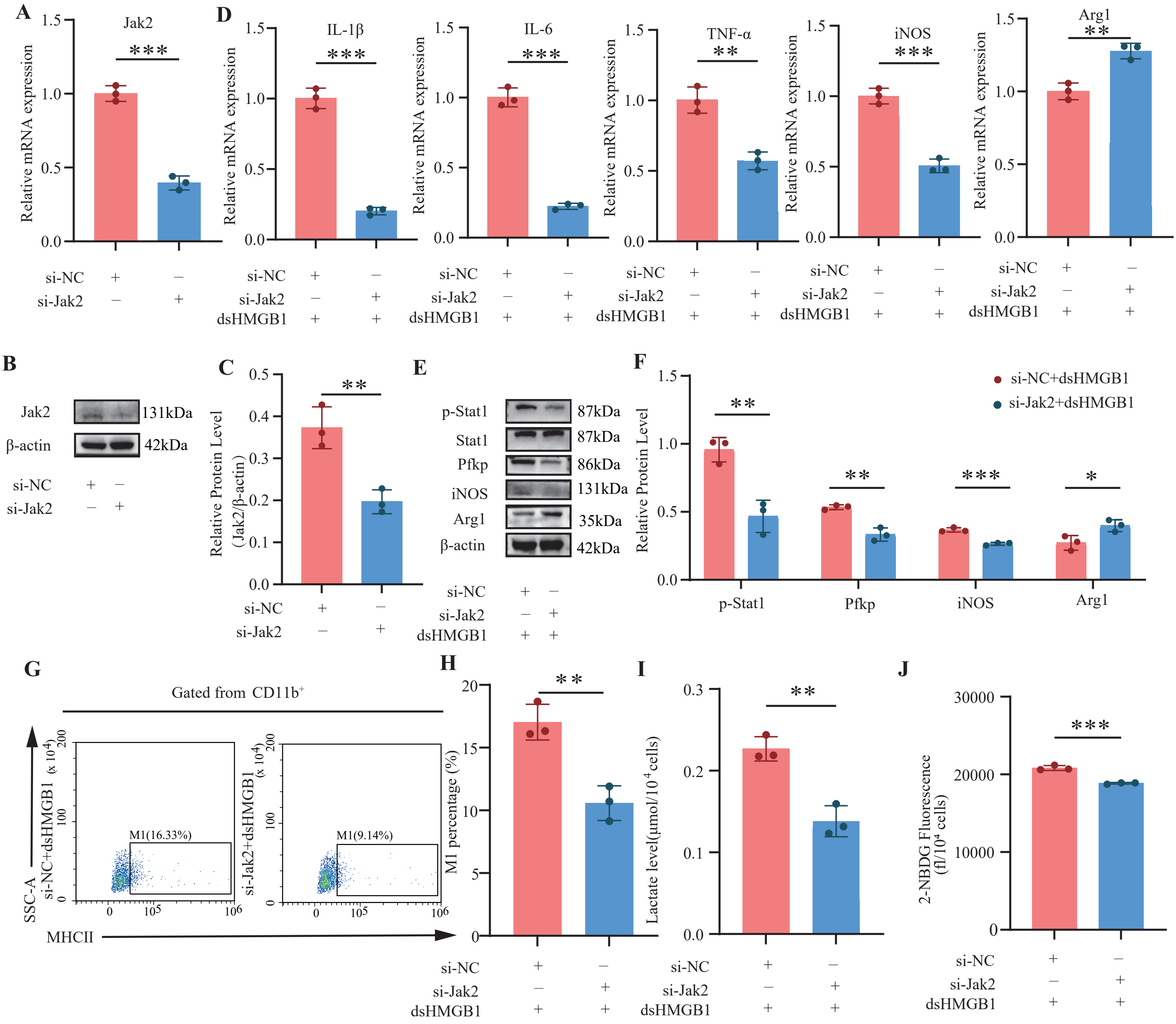

A
Jak2
***
Relative mRNA expression
si-NC
si-Jak2
D
IL-1β
IL-6
TNF-α
iNOS
Arg1
dsHMGB1
B
Jak2
131kDa
β-actin
42kDa
C
Relative Protein Level (Jak2/β-actin)
E
p-Stat1
87kDa
Stat1
87kDa
Pfkp
86kDa
iNOS
131kDa
Arg1
35kDa
β-actin
42kDa
F
Relative Protein Level
si-NC+dsHMGB1
si-Jak2+dsHMGB1
G
Gated from CD11b+
SSC-A
si-NC+dsHMGB1 (x 10^4)
si-Jak2+dsHMGB1 (x 10^4)
M1(16.33%)
M1(9.14%)
MHCII
H
M1 percentage (%)
I
Lactate level(μmol/10^4 cells)
J
2-NBDG Fluorescence (fl/10^4 cells)

Figure S4

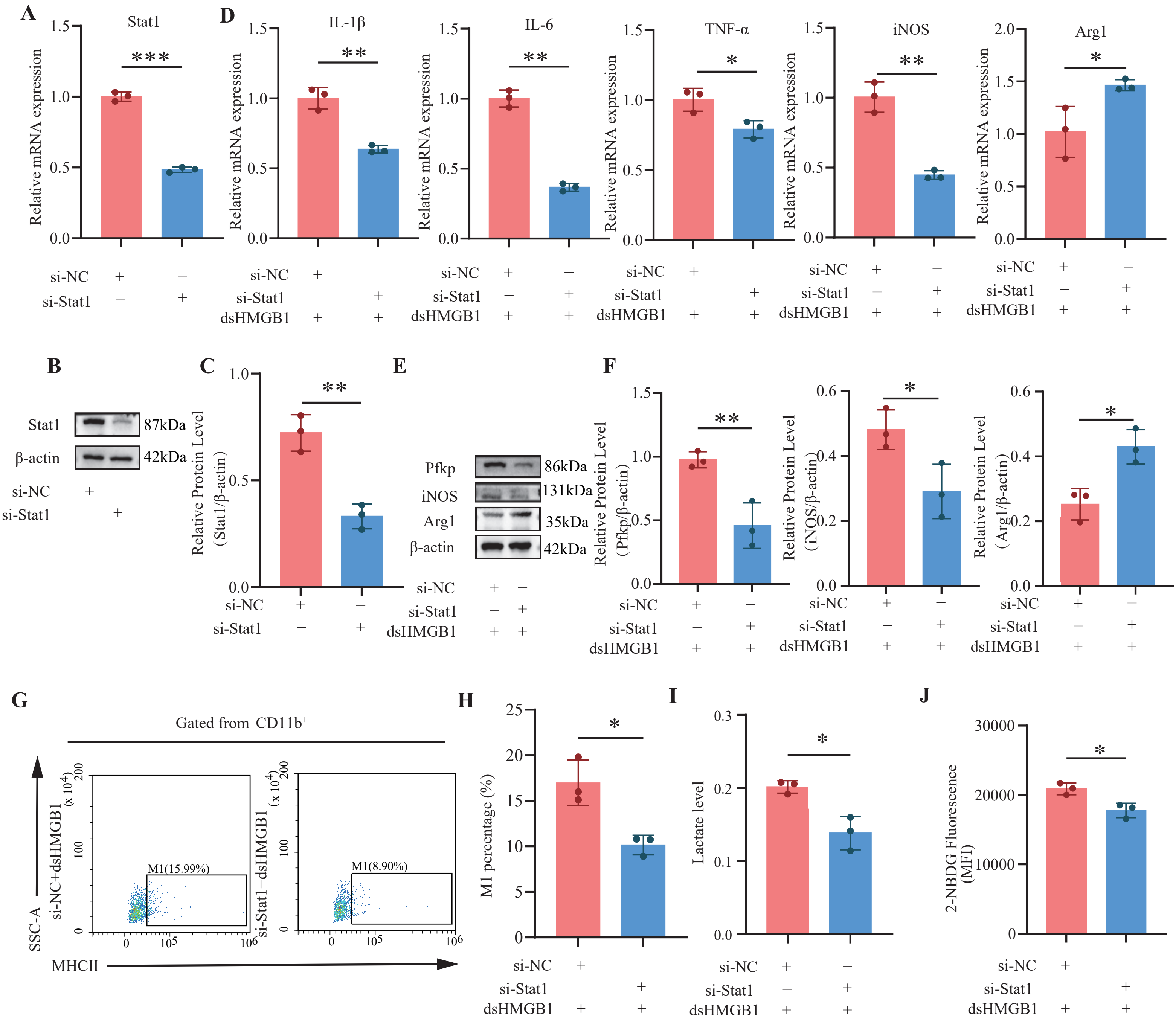
A
Stat1
Relative mRNA expression
***
si-NC
si-Stat1
D
IL-1β
IL-6
TNF-α
iNOS
Arg1
**
*
dsHMGB1
B
Stat1
β-actin
87kDa
42kDa
C
Relative Protein Level
(Stat1/β-actin)
E
Pfkp
iNOS
Arg1
86kDa
131kDa
35kDa
F
(Pfkp/β-actin)
(iNOS/β-actin)
(Arg1/β-actin)
G
Gated from CD11b+
SSC-A
MHCII
si-NC+dsHMGB1
si-Stat1+dsHMGB1
M1(15.99%)
M1(8.90%)
H
M1 percentage (%)
I
Lactate level
J
2-NBDG Fluorescence
(MFI)
